# Ecoregion-Based Conservation Planning in the Mediterranean: Dealing with Large-Scale Heterogeneity

**DOI:** 10.1371/journal.pone.0076449

**Published:** 2013-10-14

**Authors:** Sylvaine Giakoumi, Maria Sini, Vasilis Gerovasileiou, Tessa Mazor, Jutta Beher, Hugh P. Possingham, Ameer Abdulla, Melih Ertan Çinar, Panagiotis Dendrinos, Ali Cemal Gucu, Alexandros A. Karamanlidis, Petra Rodic, Panayotis Panayotidis, Ergun Taskin, Andrej Jaklin, Eleni Voultsiadou, Chloë Webster, Argyro Zenetos, Stelios Katsanevakis

**Affiliations:** 1 Institute of Marine Biological Resources and Inland Waters, Hellenic Centre for Marine Research, Ag. Kosmas, Greece; 2 ARC Centre of Excellence for Environmental Decisions, School of Biological Sciences, The University of Queensland, Brisbane, Queensland, Australia; 3 Department of Marine Sciences, University of the Aegean, Mytilene, Lesvos Island, Greece; 4 Department of Zoology, School of Biology, Aristotle University of Thessaloniki, Thessaloniki, Greece; 5 UNEP World Conservation Monitoring Center, Cambridge, United Kingdom; 6 Ege University, Faculty of Fisheries, Department of Hydrobiology, Bornova, Izmir, Turkey; 7 MOm/Hellenic Society for the Study and Protection of the Monk seal, Athens, Greece; 8 Middle East Technical University Institute of Marine Sciences, Erdemli, Mersin, Turkey; 9 State Institute For Nature Protection, Zagreb, Croatia; 10 Institute of Oceanography, Hellenic Centre for Marine Research, Anavyssos, Greece; 11 Celal Bayar University, Faculty of Arts and Sciences, Department of Biology, Manisa, Turkey; 12 Center for Marine Research Rovinj, Ruđer Bošković Institute Zagreb, Croatia; 13 MedPAN, The Network of Managers of Marine Protected Areas in the Mediterranean, Marseille, France; 14 European Commission, Joint Research Centre, Institute for Environment and Sustainability, Water Resources Unit, Ispra, Italy; National Institute of Water & Atmospheric Research, New Zealand

## Abstract

Spatial priorities for the conservation of three key Mediterranean habitats, i.e. seagrass *Posidonia oceanica* meadows, coralligenous formations, and marine caves, were determined through a systematic planning approach. Available information on the distribution of these habitats across the entire Mediterranean Sea was compiled to produce basin-scale distribution maps. Conservation targets for each habitat type were set according to European Union guidelines. Surrogates were used to estimate the spatial variation of opportunity cost for commercial, non-commercial fishing, and aquaculture. Marxan conservation planning software was used to evaluate the comparative utility of two planning scenarios: (a) a whole-basin scenario, referring to selection of priority areas across the whole Mediterranean Sea, and (b) an ecoregional scenario, in which priority areas were selected within eight predefined ecoregions. Although both scenarios required approximately the same total area to be protected in order to achieve conservation targets, the opportunity cost differed between them. The whole-basin scenario yielded a lower opportunity cost, but the Alboran Sea ecoregion was not represented and priority areas were predominantly located in the Ionian, Aegean, and Adriatic Seas. In comparison, the ecoregional scenario resulted in a higher representation of ecoregions and a more even distribution of priority areas, albeit with a higher opportunity cost. We suggest that planning at the ecoregional level ensures better representativeness of the selected conservation features and adequate protection of species, functional, and genetic diversity across the basin. While there are several initiatives that identify priority areas in the Mediterranean Sea, our approach is novel as it combines three issues: (a) it is based on the distribution of habitats and not species, which was rarely the case in previous efforts, (b) it considers spatial variability of cost throughout this socioeconomically heterogeneous basin, and (c) it adopts ecoregions as the most appropriate level for large-scale planning.

## Introduction

Understanding the distribution of marine organisms and processes is of great importance for marine conservation planning [1]. Obtaining detailed information for all species is time consuming and costly, thus practically impossible when time or resources are limited. To address this challenge, physical data or higher-taxon approaches (e.g., identification to genera or families) have often been used as surrogates for the distribution of species richness [2], [3]. Using habitat surrogates can be a cost-effective method for the identification of priority areas for conservation in coastal ecosystems [4]. In the last decades the use of habitat surrogates in spatial prioritization has been applied both at a local and regional scale for marine systems (e.g., [5], [6]). However, in the Mediterranean Sea most prioritization initiatives have been based on the distribution of large predators, commercial or flagship species (e.g., marine mammals, sea birds) failing to adequately represent a large number of species with different distribution patterns [7]. The utility of umbrella and flagship species as surrogates for regional biodiversity has been found to be limited and hence their use in conservation planning inappropriate [8].

In order to protect marine biodiversity the European Union (EU) has identified and classified a number of marine habitat types within the Habitats Directive (92/43/EEC) that should be represented in a pan-European network of protected areas (named Natura 2000). The list of marine habitats includes sandbanks that are always slightly covered by sea water, *Posidonia oceanica* beds, estuaries, mudflats and sandflats not covered by seawater at low tide, coastal lagoons, large shallow inlets and bays, reefs, submarine structures made by leaking gases, as well as submerged or partially submerged sea caves. This list has further been expanded by the Barcelona Convention which established a List of Specially Protected Areas of Mediterranean Importance (SPAMI's List), through the Protocol on Specially Protected Areas and Biological Diversity in the Mediterranean [9]. Although some of the habitats listed by both the Habitats Directive and the Barcelona Convention can be easily mapped (e.g., estuaries, coastal lagoons, large shallow inlets, and bays) and they are protected by some of the existing instruments (e.g., Natura 2000, Emerald Network, RAMSAR sites; see the supplementary material of Micheli et al. [7] for detailed description), most submerged habitat types have not yet been comprehensively mapped in the entire Mediterranean Sea [10]. Compilation of all available data on the distribution of these habitats is a first and critical step towards effective conservation planning.

In the present study we have focused our efforts on three benthic habitats of high conservation importance: *P. oceanica* seagrass meadows, coralligenous formations, and marine caves. These habitats were selected because they have been designated as Mediterranean priority habitats by the EU Habitats Directive and/or the Barcelona Convention and a large amount of distribution information exist, albeit in a non-synthesized state.

The seagrass beds and bio-constructions of the endemic *P. oceanica* are considered a priority habitat for conservation by the EU Habitats Directive and the Barcelona Convention. *Posidonia oceanica* meadows are important nursery grounds for a large number of fish and invertebrate species, thereby contributing to the maintenance of marine biodiversity [11]. Over 400 plant species and several thousands of animal species inhabit its meadows [12]. At the same time, *P. oceanica* beds are one of the most productive ecosystems on the planet; their primary production is comparable to or greater than that of tropical forests and coral reefs [12]. Seagrass meadows provide a number of ecosystem services, including food provision, coastal protection, carbon sequestration, water purification, ocean nourishment, and life cycle maintenance [13]. Nonetheless, they are among the most threatened coastal ecosystems on earth with a global decline rate of 110 km^2^ yr^−1^ since 1980 [14]. However, this number should be considered with caution as differences in mapping techniques can lead to an overestimate of the actual meadows regression [15]. Coastal development, pollution, trawling, fish farming, mooring, dredging, dumping of dredge spoil, and introduced species are the major factors responsible for the loss of *P. oceanica* meadows, and climate change further exacerbates the effects of local threats [16], [17]. Due to its very slow growth (2 cm yr^−1^; [18]), *P. oceanica* recovery and recolonization may take centuries depending on the severity of impacts. Regression of seagrass meadows results in decline of the services they provide, emission of vast quantities of stored carbon, decline in the distribution range of associated species, and disruption of critical linkages with other habitats.

Coralligenous formations are the second most diverse benthic habitat of the Mediterranean Sea after *P. oceanica* meadows [19] that are included in the EU Habitats Directive under the generic habitat type “Reefs”. Furthermore, an Action Plan has been adopted by contracting parties of the Barcelona Convention specifically aiming at their conservation [20]. Coralligenous formations comprise various benthic assemblages, which form typical underwater seascapes in the sublittoral zone [20]. Coralline algal frameworks growing in dim light conditions are their main components, although it is the presence of a broad range of sciaphilic and perennial organisms that characterize these complex structures and greatly contribute to their development [21]. Conservative estimates list more than 1700 species inhabiting the coralligenous assemblages (15–20% of Mediterranean species), among which are several protected and commercially important species [21]–[23]. Also known for their high aesthetic value, coralligenous structures constitute focal points for underwater tourism and recreational diving. Their extensive distribution, structural complexity, species diversity, role in energy flux and carbon cycle, and economic value render coralligenous structures as one of the most important coastal habitats in the Mediterranean [21], [24]. Currently, they are among the most threatened habitats in the region; as key engineering species they are long-lived with slow growth rates, while the dynamic equilibrium between the bio-construction and bio-erosion processes is particularly susceptible to environmental changes [25]–[28]. Direct or indirect human-induced disturbances include mechanical damage mainly caused by destructive fishing practices, pollution, sedimentation, diver frequentation, biological invasions, mass mortality outbreaks related to temperature anomalies, and the synergistic effects of these stressors [29]–[33].

Coralligenous rims are commonly formed at the entrance zone of “submerged or partially submerged sea caves” that are protected by the EU Habitats Directive as a distinct habitat type. Semi-dark caves are also included in the aforementioned Action Plan regarding the coralligenous and other calcareous bio-concretions [20], while an additional Action Plan dedicated to the conservation of dark habitats, encompassing dark caves and deep-sea habitats (e.g., deep-sea corals), has recently been developed [34]. As each cave system is characterized by a unique topographical complexity and associated abiotic gradients, marine caves host a variety of communities. These range from semi-sciaphilic and coralligenous algal-dominated assemblages to semi- and entirely-dark assemblages [35], which in some cases resemble those of the deep sea [36], [37]. Mediterranean marine caves harbour a high number of rare, endemic, protected, and commercially important species such as the red coral *Corallium rubrum*. The survival of the Mediterranean monk seal *Monachus monachus*, which is a critically endangered species, has been favoured by a plethora of suitable caves for resting and pupping predominantly in the eastern Mediterranean Sea [38]. Marine caves have been characterized as ‘refuge habitats’ [39], ‘ecological islands’ supporting isolated populations [39], [40], ‘bathyal mesocosms’ within the littoral zone [37], and ‘biodiversity reservoirs’ [41]. Furthermore, they present high aesthetic, and often archaeological value, offering popular sites for SCUBA diving activities. Nonetheless, marine caves are ecosystems with low resilience [39] that are sensitive to diver-induced mechanical disturbance, impacts of increasing water temperature on motile and sessile invertebrates, red coral harvesting, coastal infrastructure and development, and marine pollution ([42]–[45], authors' personal observations).

Although all three habitat types under study are to some extent represented in Mediterranean Marine Protected Areas (MPAs), they are not adequately protected [46]. This limited protection is due to either their low percentage of coverage within existing MPAs, or because most MPAs lack a management structure or effective management plan. This is particularly the case in the southern part of the Mediterranean Sea, since 96% of current MPAs (Natura 2000 sites included) are situated in the northern Mediterranean basin [46]. Recently, scientific consortia have focused on raising public awareness on the urgent need for increased biodiversity protection in the Mediterranean Sea, especially in poorly protected regions (e.g., [7], [47], [48]). These scientific initiatives come to support the Antalya declaration and the Roadmap to 2020 established during the Forum for Mediterranean MPAs (held in November 2012 in Turkey). During the Forum, the Mediterranean MPA community reviewed the status of MPAs in the region and identified the actions needed for the establishment of an ecological network of MPAs and its effective and sustainable management. A roadmap was produced calling for urgent action and aimed at achieving the conservation objectives set by international commitments by 2020.

Adequate representation of the three examined habitats, especially coralligenous formations and marine caves, in existing Mediterranean MPAs or proposed conservation plans [7] has been hindered by the substantial heterogeneity of their “known” distribution. Such heterogeneity encompasses two underlying sources of variability: (1) the uneven natural distribution of the three habitats due to the spatial patterns of related geophysical and oceanographic conditions [23], [49], and (2) the highly variable mapping and monitoring efforts across the Mediterranean basin [50]. Due to this heterogeneity, not all ecoregions, i.e. areas of relatively homogenous species composition that are clearly distinct from adjacent systems [51], will be adequately represented in large-scale whole-basin conservation plans when using habitats as surrogates for the distribution of biodiversity. Similar heterogeneity issues affect large-scale conservation planning when the process is based on the spatial distribution of a restricted number of species or higher taxa (see [23], [47]).

The use of biogeographic classification can support effective and representative marine conservation that protects the full range of biodiversity (genes, species, and ecosystems) [51]–[53]. Representation of *P. oceanica* beds, coralligenous formations, and marine caves in different ecoregions is crucial from both a functional and genetic point of view [54]. While *P. oceanica* exhibits low genetic variability across the basin [12], the composition of communities hosted in its meadows differs among ecoregions [55], [56]. Similarly, invertebrate assemblages constructing coralligenous habitats and inhabiting marine caves vary significantly among or even within ecoregions [41], [57], [58]. For effective protection of various aspects of biodiversity, a more even spatial distribution of MPAs is needed across the Mediterranean Sea [59].

Spatial variability is not only a characteristic of biodiversity, but also of anthropogenic activities taking place in the human-dominated environment of the Mediterranean Sea [60], [61]. The application of systematic conservation planning requires the inclusion of socioeconomic cost so that the plans proposed are feasible [62], [63]. However, spatially explicit economic information is often unavailable [62] or its resolution is inadequate [64]. In such cases, spatially variable cost surrogates should be used rather than assuming just area coverage as a surrogate for cost [65]. Taking into account opportunity cost for fisheries, which is the most prevalent activity in the sea [66], can lead to win-win situations and support equitable and efficient conservation planning.

The present study aims at identifying priority areas for the conservation of the seagrass *P. oceanica*, coralligenous formations, and marine caves across the entire Mediterranean Sea (excluding areas deeper than 1000 m), considering concurrent ecoregional representation of habitats and opportunity cost. The selection of priority areas within eight marine ecoregions [67] was based on a systematic planning process: i) distribution maps for the habitats under study were produced following a thorough assimilation of available data, ii) surrogates for the spatial distribution of opportunity cost for commercial fishing, non-commercial fishing, and aquaculture were used, and iii) the systematic computational tool Marxan was applied. Potential conservation actions within the resulting priority areas are further discussed, given that the primary focus of conservation planning is the prioritization of actions rather than places [68]. This approach is novel in large-scale multinational conservation planning, especially in the Mediterranean environment (see [7]).

## Methods

### Study area

**Figure 1:**
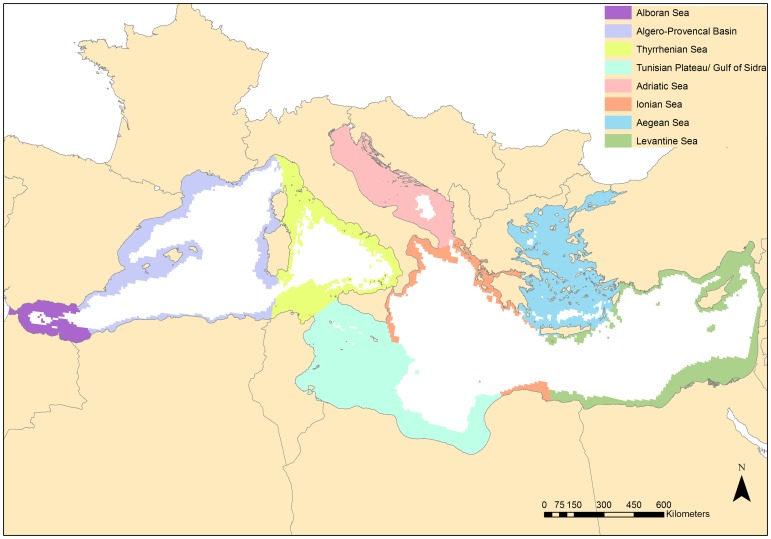
Map of the study area divided into 8 ecoregions, *sensu* Notarbartolo di Sciara and Agardy (2010). The study area comprises the entire Mediterranean Sea, excluding areas deeper than 1000

The study area comprises the entire Mediterranean Sea, excluding areas deeper than 1000 m (Fig. 1). We did not include areas at depths beyond 1000 m in our analysis because (1) the three targeted habitats generally thrive at much shallower depths (with the exception of some rare occurrences of very deep corals), (2) human activities and their impacts to marine biodiversity are predominantly concentrated on the continental shelves and slopes of the basin [47], and (3) decisions have already been made for their protection. The General Fisheries Commission for the Mediterranean (GFCM) recommended the prohibition of towed dredges and trawl nets fisheries at depths beyond 1000 m (Recommendation GFCM/2005/1 on the “management of certain fisheries exploiting demersal and deepwater species”) and the EU has adopted this recommendation through Regulation 1967/2006. Furthermore, Ecologically and Biologically Significant Areas (EBSAs) have already been identified in the Mediterranean Sea for the protection of pelagic and off-shore habitats [67]. Within these large areas, representative networks of MPAs will be established. EBSAs have been discussed, amended, and ultimately endorsed by all the contracting parties to the Barcelona Convention (21 Mediterranean countries and the EU) [69].

We divided the study area into 13212 planning units each of 10×10 km. This resolution was chosen following EU guidelines on the use of a pan-European 10×10 km grid for spatial planning (Directive 2007/2/EC). All planning units were assigned to one of the eight marine ecoregions proposed by Notarbartolo di Sciara and Agardy [67] for the identification of the EBSAs in the Mediterranean Sea: 1. Alboran Sea, 2. Algero-Provencal Basin, 3. Tyrrhenian Sea, 4. Tunisian Plateau/Gulf of Sidra, 5. Adriatic Sea, 6. Ionian Sea, 7. Aegean Sea (including the Sea of Marmara), and 8. Levantine Sea (Fig. 1). This classification is based on the work of Spalding et al. [51] with further subdivision of the western basin into two regions to capture more spatial heterogeneity. Although there is controversy on the ecological meaning of subdividing open ecosystems (such as marine ecosystems), the common geo-morphological features and ecological processes taking place into each of the eight sub-regions have led to a general consensus in their acceptance (see [69]). The boundaries among these ecoregions lack precision and are not politically sanctioned [69]; for the purpose of our analysis we have made them discrete by attributing each planning unit to a specific ecoregion (Fig. 1).

### Conservation features

Information on the distribution of the seagrass *P. oceanica* meadows, coralligenous formations, and partially or totally submerged marine caves was compiled from several source types for the production of distribution maps. As the Mediterranean scientific community has not yet reached a consensus on the exact definition of the coralligenous habitat, we consider the clarification of the data incorporated in our analyses important. The coralligenous habitat data set includes all benthic assemblages thriving on hard substrates of biogenic origin under low irradiance levels that are most commonly observed between 20–120 m depth [33]. However, particular instances of other calcareous bio-concretions, such as rhodolith beds in coastal detritic bottoms, certain parts of deep sea habitats (e.g., seamount peaks, off-shore rocky banks) characterized by high density of suspension feeders, and the rare occurrence of deep coral communities, found on seabed shallower than 1000 m, were incorporated in this data set. The inclusion of such sites was considered appropriate, as they are either found in close association to coralligenous formations or their communities share certain common characteristics with those of the deep coralligenous communities, at least with regard to the main habitat forming biotic components.

To map the spatial distribution of habitats we used the following data sources: scientific and grey literature (including journal articles, monographs, presentations and posters in conferences and workshops, reports), on-line databases and national catalogues (provided by national or international, governmental, intergovernmental-EU agencies and non-governmental organizations-NGOs), unpublished data provided by scientific officers and researchers affiliated with universities, research institutes, NGOs and governmental agencies, situated in several Mediterranean countries (including the authors), published (in the form of booklets and diving guides) and unpublished information provided by diving and caving clubs, divers and cavers through scientific and naturalist fora on the web, and direct personal communications. A complete list of all sources used for each habitat type is presented in detail in the Supplementary Online Material (Text S1). Moreover, researchers, divers, cavers and naturalists that provided information are acknowledged in the relevant section. Spatial information on the habitats was extracted or provided in the form of coordinates, Google Earth placemarks, maps (imported and digitized through ArcGIS 10), and GIS layer files. Following EU recommendations [70], habitat data were projected into the ETRS89 Lambert Azimuthal Equal Area coordinate reference system at the selected planning unit scale, using ArcGIS 10 software. To deal with the high heterogeneity in data format (points, lines or polygons) and resolution/accuracy of available data for *P. oceanica* beds and coralligenous formations, and to achieve large-scale integration of the compiled dataset, we transformed all spatial information to presence/absence in the 10×10 km planning units of our standard grid. The total number of marine caves was estimated for each planning unit.

### Socioeconomic data

The cost surrogate layer used in the analyses was developed by Mazor et al. (unpublished data) refining methods devised in Mazor et al. [71]. It represents the spatial distribution of the combined opportunity cost for three marine sectors: commercial (small and large-scale) fishing, non-commercial fishing (recreational and subsistence), and aquaculture. The opportunity cost in this study, is the lost revenue that would occur by the restriction of activities from these marine sectors when an area is designated as MPA. Estimation of opportunity cost for commercial fishing was based on annual tonnage data regarding total fishing from 28 Geographical Sub-Areas (GSAs) as reported by the GFCM for 2008 [72]. Each planning unit was assigned to one of the 28 GSA regions. It was assumed that the opportunity cost is proportional to the size of the nearest port and decreases exponentially with distance from port. To ensure that the total value of catch in each region (28 GSA regions) summed to its real value the cost of commercial fishing was normalized in each planning unit by a measure of total regional effort. To obtain a final value the fishing effort in each planning unit was multiplied by the average market value (€ per kg, http://en.fishprices.net/home; [73]) of the main species composing the catch of each fishing sector (see [74]–[76]).

Estimation of opportunity cost for non-commercial fishing was based on the number of recreational fishers per country, the cost of expenditure on fishing gear (adjusted for each country based on purchasing power parity (PPP) rates; http://data.worldbank.org/indicator/PA.NUS.PPPC.RF), and the value of catch per year. Those variables were based on the limited available information on recreational fishing in the Mediterranean Sea [77]–[79]. The cost of expenditure was used to estimate the value that recreational fishers give to recreational fishing through their purchases in the related markets, e.g., recreational vessel purchases from fishers participating in this activity through their revealed preference (hedonic method; see [77]). The value of the catch was calculated by multiplying the number of fishing days per year, the total number of kg of fish per day and the value of catch (€ per kg). Although recreational fishers are not allowed to sell their catches in many Mediterranean countries, the consumption of their catch constitutes a benefit (i.e. the avoided cost for subsistence).

The opportunity cost for aquaculture was based on the work of Trujilo et al. [80] and the factors included were the annual aquaculture production for each country, each country's sea bream (*Sparus aurata*)/sea bass (*Dicentrarchus labrax*) ratio (as those are the main fish species cultivated), the market value of the sea bream and sea bass, and the spatial distribution and area coverage of each aquaculture unit. All methods applied for the creation of cost surrogates and the importance of using cost in large-scale conservation planning are thoroughly analyzed and discussed in a separate paper recently submitted by Mazor et al.

### Spatial conservation prioritization

To select priority areas for conservation features that minimize conflict between conservation objectives and sectors of marine resources exploitation we used the conservation planning software Marxan [81]. This software uses a simulated annealing algorithm to find a range of good near-optimal systems of priority areas that meet conservation targets while attempting to minimize socioeconomic costs. Marxan solutions are generated iteratively by randomly changing the status of a single planning unit and assessing the new configuration in terms of achieving Marxan's goal, i.e. minimize the cost of the reserve network and the boundary length of the system whilst meeting a set of biodiversity targets. In Marxan the user needs to set a target for every feature to be protected which in our case was expressed as the percentage of its extent; 60% for *P. oceanica* and 40% for the other two habitat types. These targets were based on the EU additional guidelines for assessing sufficiency of Natura 2000 proposals (SCIs) for marine habitats and species [82]. In the guidelines of the European Topic Centre on Biological Diversity it is stated: “Where quantitative data on habitat areas are available, it would be possible to apply the arbitrary sufficiency levels 20–60% for non-priority habitats and >60% for priority habitats (e.g., *Posidonia* beds)”. Although coralligenous and marine caves are considered priority habitats under the Barcelona Convention, the EU formally recognizes only *P. oceanica* meadows as a priority habitat. Using the same targets we produced two planning scenarios: a) selection of priority areas across the whole Mediterranean Sea, and b) selection of priority areas across each ecoregion separately.

For our priority areas to have the desired level of spatial compactness we calibrated the Boundary Length Modifier (BLM) to generate a reasonable trade-off between boundary length and cost [83]. After several trials and calibration of our model, we found that using a BLM value of 100 produced solutions with a desirable level of compactness (i.e. selected planning units were not scattered all over the study area but were sufficiently clustered with a reasonable trade-off with cost). Marxan was run 1000 times. By using the selection frequency, which is the proportion of runs in which a site (planning unit) is selected amongst the 1000 runs, we defined the areas of greater irreplaceability and hence higher priority for protection.

Finally, the spatial distribution of the priority areas identified in the ecoregion scenario was compared to the distribution of 677 existing MPAs, including national MPAs, Natura 2000 sites, and SPAMIs. Data on current MPAs distribution was provided by MAPAMED; The database on Mediterranean Marine Protected Areas [84]. The MPA definition by MAPAMED is: “MPA is a clearly defined marine geographical space – including subtidal, intertidal and supratidal terrain, and coastal lakes/lagoons connected permanently or temporally to the sea, together with its overlying water – recognized, dedicated and managed, through legal or other effective means, to achieve the long-term conservation of nature with associated ecosystem services and cultural values”.

The use of presence/absence data and the coarse resolution used in our analyses (10×10 km grid) did not allow us to perform a detailed coverage assessment of the spatial overlap between the priority areas identified and current MPAs. Hence, we calculated the percentage of planning units for each ecoregion that: a) did not contain an MPA but was identified as priority area, b) contained an MPA and was identified as priority area, c) did not contain an MPA and was not identified as a priority area, and d) contained an MPA but was not identified as priority area.

## Results

### Habitat distributions

**Figure 2:**
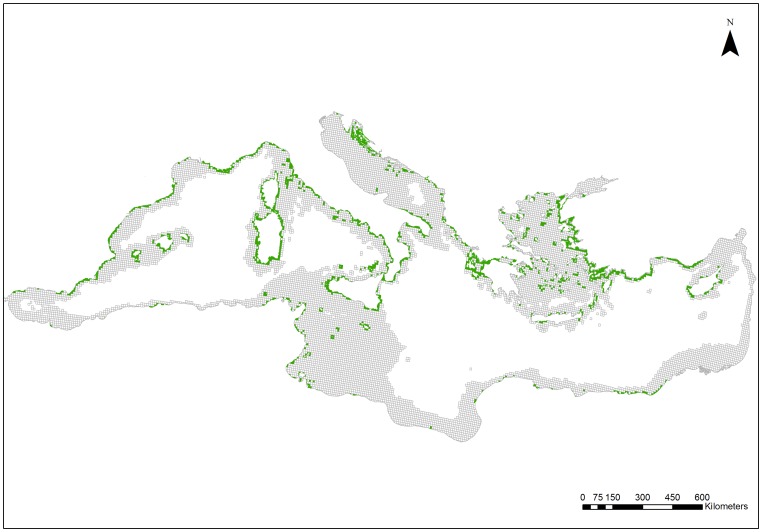
Distribution of *Posidonia oceanica* meadows in the Mediterranean Sea. Green cells indicate planning units with the presence of *P. oceanica*. Information on the spatial distribution of *P. oceanica* was extracted from various sources which are provided as a supplementary material.

The compilation of data on the distribution of *P. oceanica* meadows (see Text S1) is illustrated in Fig. 2. *Posidonia oceanica* meadows have been reported in 16 Mediterranean countries, i.e. Albania, Algeria, Croatia, Cyprus, Egypt, France, Greece, Italy, Libya, Malta, Monaco, Montenegro, Morocco, Spain, Tunisia, and Turkey (Fig. 2; Text S1). Availability of data was greater in the northern part of the Mediterranean than in the southern part. The Aegean Sea presented the highest coverage in terms of absolute numbers of planning units with presence of *P. oceanica* meadows, followed by the Algero-Provencal Basin and the Tyrrhenian Sea (Table 1). The Ionian, Levantine, and Adriatic Seas had intermediate coverage while the Tunisian Plateau/Gulf of Sidra and the Alboran Sea had the lowest. However, the relative *P. oceanica* coverage within each ecoregion (% of planning units with presence of *P. oceanica* meadows) was greater in the Ionian Sea, as well as in the Tyrrhenian Sea and the Algero-Provencal Basin (Table 1). Very low relative coverage was found in the Alboran Sea and the Tunisian Plateau/Gulf of Sidra.

**Table 1:** Distribution of habitats across ecoregions of the Mediterranean Sea.

Ecoregion	Planning Units	*P. oceanica*			Coralligenous			Marine Caves				
	N	Ns	N %	Nr %	Nc	N %	Nr %	Nmc	N %	Nr %	Nc	Nc %
**Alboran Sea**	496	16	0.1	3	25	0.2	5	6	0.05	1.2	6	0.2
**Algero-Provencal Basin**	1747	370	2.8	21	153	1.2	9	111	0.84	6.4	459	16
**Tyrrhenian Sea**	1570	339	2.6	22	171	1.3	11	78	0.59	5	581	20.3
**Tunisian Plateau/Gulf of Sidra**	2975	119	0.9	4	48	0.4	2	12	0.09	0.4	68	2.4
**Adriatic Sea**	1485	151	1.1	10	276	2.1	19	181	1.37	12.2	708	24.7
**Ionian Sea**	791	228	1.7	29	88	0.7	11	58	0.44	7.3	307	10.7
**Aegean Sea**	2423	408	3.1	17	250	1.9	10	184	1.39	7.6	529	18.5
**Levantine Sea**	1725	161	1.2	9	64	0.5	4	77	0.58	4.5	209	7.3

We calculated (i) the number of planning units in each ecoregion (N); (ii) the number of planning units with presence of each habitat in each ecoregion (*P. oceanica*: Ns, coralligenous: Nc, marine caves: Nmc), (iii) the percentage of Ns, Nc, Nmc across the Mediterranean Sea (N %), (iv) the percentage of Ns, Nc, Nmc across each ecoregion (Nr %); (v) the number of caves in each ecoregion (Nc) and (vi) the percentage of marine caves of each ecoregion across the Mediterranean Sea (Nc %).

**Figure 3:**
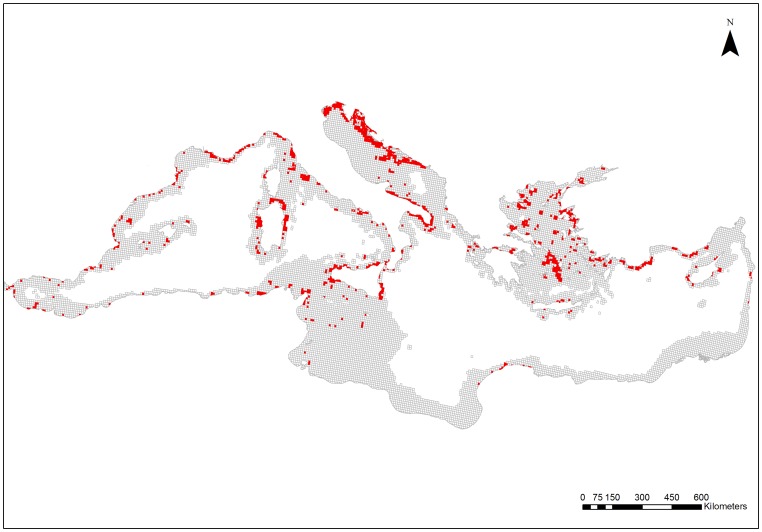
Distribution of coralligenous formations in the Mediterranean Sea. Red cells indicate planning units with the presence of coralligenous formations. Information on the spatial distribution of coralligenous formations was extracted from various sources which are provided as a supplementary material.

The distribution of coralligenous formations, based on the review of available information (see Text S1), is depicted in Fig. 3. Coralligenous habitats have been recorded in 16 Mediterranean countries, i.e. Albania, Algeria, Croatia, Cyprus, France, Greece, Italy, Israel, Lebanon, Libya, Malta, Monaco, Morocco, Spain, Tunisia, and Turkey. Information sources were substantially greater for the northern than the southern part of the Mediterranean. The Adriatic and Aegean Seas presented the highest coverage in terms of absolute numbers of planning units with presence of coralligenous formations, followed by the Tyrrhenian Sea and the Algero-Provencal Basin (Table 1). All other ecoregions presented lower coverage, with the Alboran Sea having the lowest. The relative coverage of coralligenous habitats within each ecoregion (% of cells with presence of coralligenous) was the highest in the Adriatic Sea, followed by the Tyrrhenian, Ionian, and Aegean Seas, while it was much lower in the north-eastern parts of the Levantine Sea and the Tunisian Plateau/Gulf of Sidra (Table 1).

**Figure 4:**
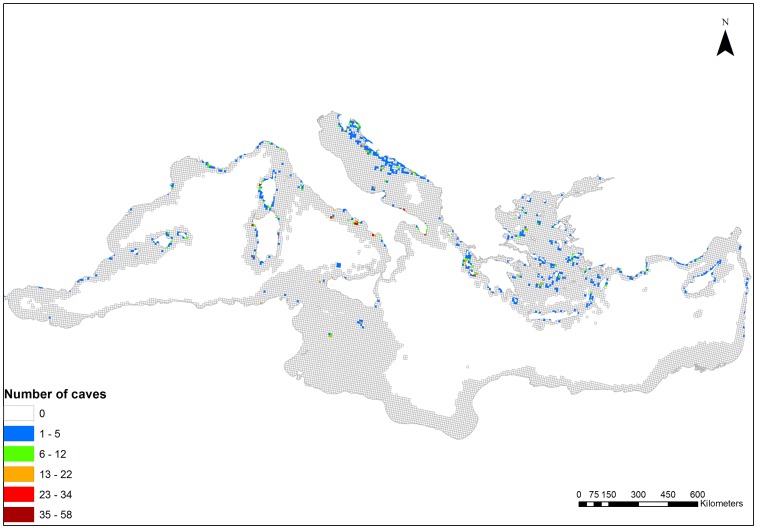
Distribution of marine caves in the Mediterranean Sea. Different colours indicate the variation in cave number per planning unit, warmer colours illustrating planning units with higher number of caves. Information on the spatial distribution of marine caves was extracted from various sources which are provided as a supplementary material.

Almost 3000 marine caves were recorded in 14 Mediterranean countries, i.e. Albania, Croatia, Cyprus, France, Greece, Israel, Italy, Lebanon, Malta, Montenegro, Morocco, Spain, Tunisia, and Turkey. The distribution of marine caves in the Mediterranean basin is illustrated in Fig. 4, while sources of data are listed in Text S1. The vast majority (about 97%) of caves recorded was located in the northern Mediterranean basin. The Aegean Sea presented the highest coverage in terms of absolute numbers of planning units with presence of marine caves, followed by the Adriatic Sea and the Algero-Provencal Basin (Table 1). The highest number of caves was found in the Adriatic Sea, followed by the Tyrrhenian Sea, the Aegean Sea, and the Algero-Provencal Basin. The Ionian and Levantine Seas had intermediate numbers of marine caves, while the Tunisian Plateau/Gulf of Sidra and the Alboran Sea had the lowest numbers (Table 1). A dense concentration of caves (Fig. 4) within a single planning unit was found in parts of the Algero-Provencal Basin (e.g., northwest Corsica), Tyrrhenian (e.g., Pontine Islands and Capo Palinuro), south-west Adriatic (Bari region), and Ionian Seas (Salento Peninsula and Zakynthos Island). Several other regions and particularly insular areas (e.g., the Greek Aegean, Croatian and Balearic islands) also presented a high number of marine caves but were more evenly distributed across the coastline.

### Cost distribution

**Figure 5:**
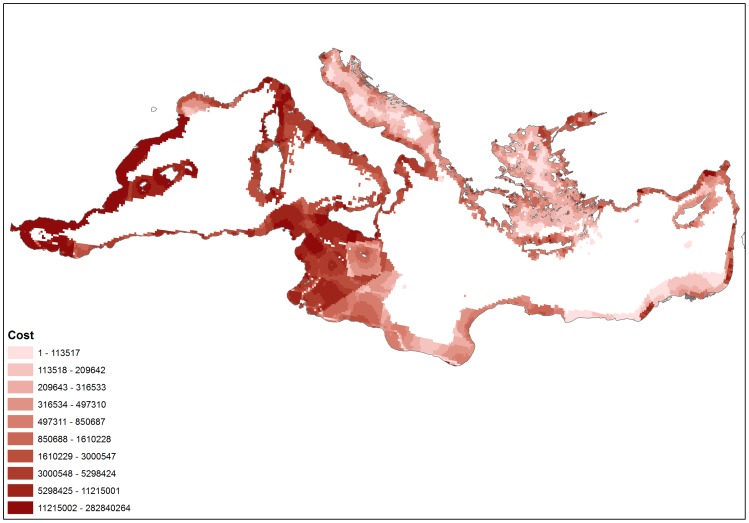
Distribution of opportunity cost for commercial fishing, non-commercial fishing and aquaculture across the Mediterranean Sea. Darker shades indicate areas with higher opportunity cost (in Euros).

Overall, the western Mediterranean Sea was found to be more expensive for conservation in terms of opportunity cost than the eastern part of the basin. Particularly high opportunity cost was computed along the Spanish coast in the Alboran Sea and Algero-Provencal Basin (Fig. 5). The high cost of the planning units is associated with the distance from the coastline, because non-commercial (recreational and subsistence) fishing and small-scale commercial efforts are concentrated close to the shore within 12 nautical mile territorial waters [85]. Furthermore, the narrow continental shelf dominating the Mediterranean also determines that most activities are concentrated near the coast, even large-scale fisheries. Thus, near-shore areas were more expensive than offshore areas throughout the study region.

### Priority areas

**Figure 6:**
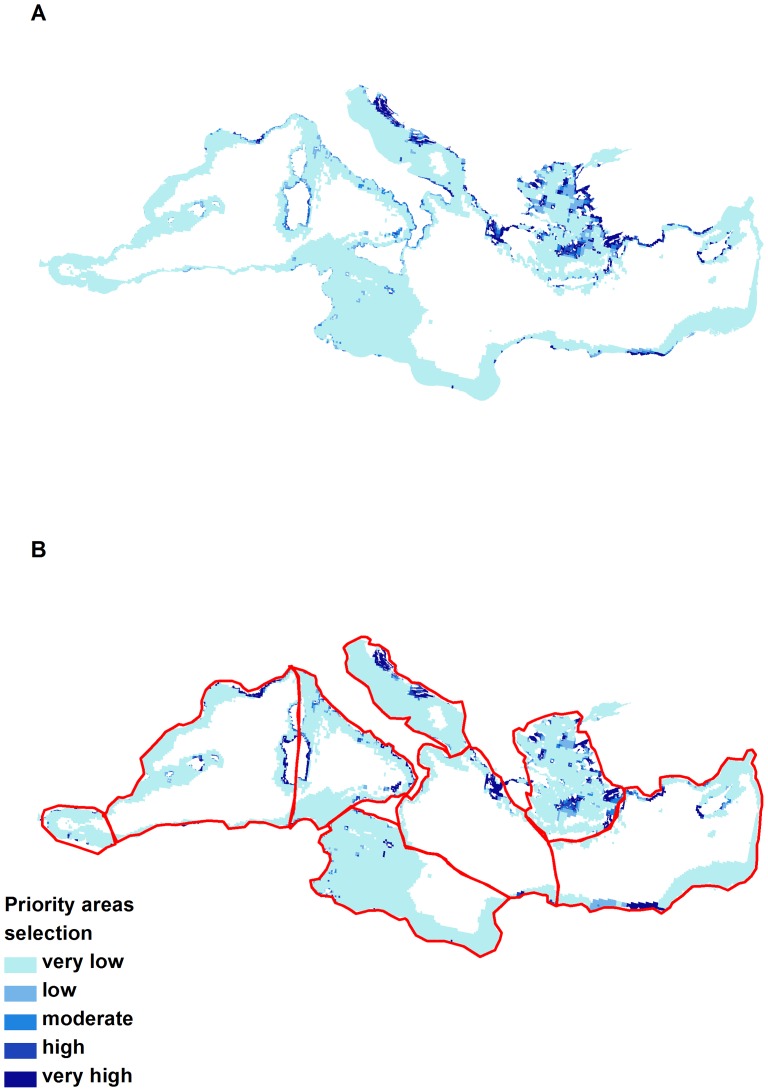
Priority conservation areas when planning a) at whole basin scale and b) at ecoregional scale. Darker shades correspond to areas with higher selection frequency and therefore constitute spatial priorities. Red delineation illustrates borders of the 8 ecoregions.

In the whole-basin planning scenario (targets set for the entire Mediterranean Sea), higher priorities were mostly located in: a) the Greek Ionian Archipelago and Patraikos Gulf, b) the Aegean Sea, particularly in the Cyclades Archipelago and along the Turkish coast, and c) the Adriatic Sea along the Croatian coast. According to this scenario, 25% of the Ionian, 23% of the Aegean, and 15% of the Adriatic Sea constitute areas highly selected as conservation priorities (planning unit selection frequency >50%), whereas no area was selected in the Alboran Sea (Fig. 6A; Table 2).

**Table 2:** Percentage of area cover and opportunity cost when planning at different spatial scales.

Ecoregion	Area %		Cost %	
	Scenario a	Scenario b	Scenario a	Scenario b
**Alboran Sea**	0	4	0	3
**Algero-Provencal Basin**	5	14	0.4	3
**Tyrrhenian Sea**	8	14	2	5
**Tunisian Plateau/Gulf of Sidra**	1	3	0.3	2
**Adriatic Sea**	15	12	12	6
**Ionian Sea**	25	23	10	7
**Aegean Sea**	23	17	10	6
**Levantine Sea**	11	9	6	3
**Mediterranean Sea**	10.76	11.25	1.45	3.41

Percentage area cover and percentage opportunity cost of planning units required in each ecoregion of the Mediterranean Sea for scenarios a and b (scenario a: selection of priority areas across the whole Mediterranean Sea, and b: selection of priority areas for each ecoregion separately).

In the ecoregion scenario (targets set for each ecoregion separately), the Ionian, Aegean, and Adriatic Seas remained high priorities but to a lower extent (Fig 6B; Table 2). On the other hand, the proportion for protection increased in the Algero-Provencal Basin and the Tyrrhenian Sea by 180% and 75% respectively, while 4% of the Alboran Sea was identified as priority for conservation. Both scenarios were equally demanding in terms of total area (approximately 11% of the planning units of the study area). However, the translocation of priority areas from cheaper to more expensive areas in this scenario increased the overall cost of protection by a factor of approximately 2.5 (Table 2).

**Figure 7:**
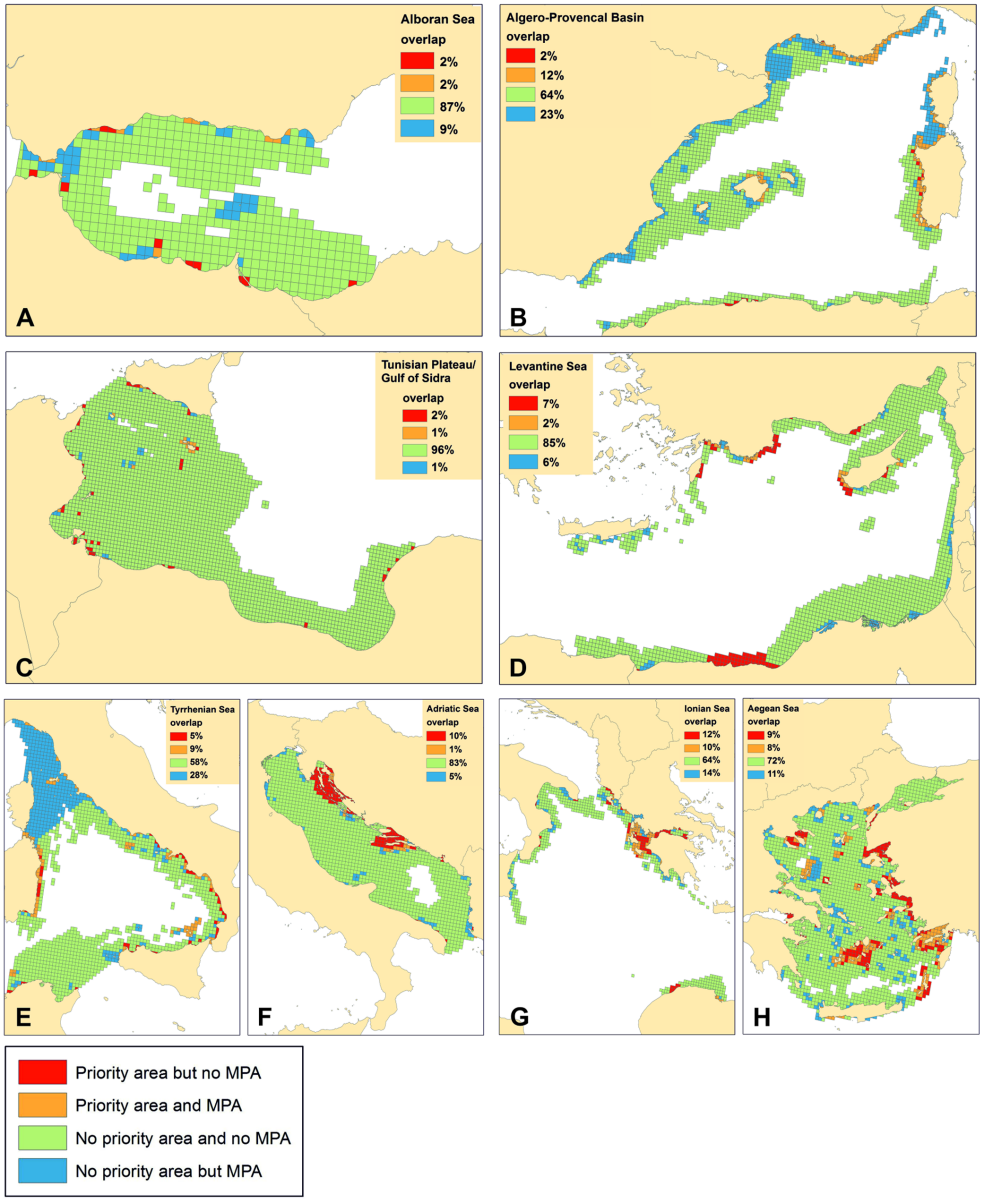
Spatial overlap between the identified priority conservation areas and existing MPAs in each ecoregion. Different colours have been used to illustrate the percentage of planning units for the A. Alboran Sea, B. Algero-Provencal Basin, C. Tunisian Plateau, D. Levantine Sea, E. Tyrrhenian Sea, F. Adriatic Sea, G. Ionian Sea, and H. Aegean Sea, that a) did not contain an MPA but was identified as priority area (red), b) contained an MPA and was identified as priority area (orange), c) did not contain an MPA and was not identified as a priority area (green) and d) contained an MPA but was not identified as priority area (blue).

The spatial overlap between priority areas identified in the ecoregion scenario and current MPAs was more pronounced in the Algero-Provencal Basin (12% of the ecoregion planning units), the Ionian Sea (10%) and the Tyrrhenian Sea (9%; Fig. 7). Less overlap was observed in the Adriatic Sea (1%) and the Tunisian Plateau/Gulf of Sidra (1%; Fig. 7). A significant amount of areas identified as priority areas in the Ionian (12%), Adriatic (10%) and Aegean Seas (9%) have no protection status. On the other hand, areas under legal protection in the Tyrrhenian Sea (28%) and the Algero-Provencal Basin (23%) were not identified as priority areas for the habitats under study.

## Discussion

Delay in achieving the Aichi goals set by the Convention on Biological Diversity, specifically Target 11 committing states to protect 10% of the Mediterranean Sea by 2020, has often been attributed to the lack or scarcity of data on biodiversity distribution [7], [69]. In the present study, we compiled all available information for three Mediterranean habitats of conservation concern, namely *P. oceanica* meadows, coralligenous formations, and marine caves and identified priority areas for their conservation. When planning at a whole-basin scale, priority areas were mostly concentrated in the Ionian, Aegean, and Adriatic Seas due to the high occurrence of these three habitat types and the relatively low opportunity cost. When planning and setting targets for our conservation features at an ecoregional scale, the total required percentage area for protection remained the same (approx. 11%), but the priority areas identified were more evenly distributed across the Mediterranean Sea. However, while this prioritization was spatially more uniform it had higher opportunity cost, making it less cost efficient than the whole-basin plan. These results are in accordance with the findings of previous studies, where the trade-off between cost and planning scale was also observed [71], [86].

Despite the fact that planning at an ecoregional scale may increase the overall opportunity cost for the exploitation sectors of living marine resources, representation of all Mediterranean ecoregions is desirable from both a biodiversity and social point of view [87]. Representativeness at the ecoregional scale will ensure the conservation of species, functional, and genetic diversity across the Mediterranean Sea [54]. Yet, it is probable that the ecoregional classification used for the identification of EBSAs and adopted in the present study is not sufficient for the protection of habitats that present great differences in biotic diversity among ecoregions [41]. In such cases, further subdivision of ecoregions might be needed (see [88], [22]). No matter what subdivision pattern has been followed, the achievement of conservation targets in each ecoregion increases the resilience of the ecosystems to ongoing environmental and human induced changes in the Mediterranean Sea, such as climate change and the introduction of alien species [89], [90]. Additionally, a more even distribution of priority areas across the basin is more likely to provide equitable social outcomes, i.e. equitable distribution of benefits and costs across the Mediterranean coastal communities [91].

Historically, long-term research focusing on the examined habitat types in the north-western and northern Mediterranean countries could justify, at least to some extent, the observed distribution patterns. Several habitat mapping studies of *P. oceanica* and coralligenous beds (to a smaller extent) have been undertaken, mainly in MPAs (e.g., [24], [92], [93]). At the same time, there has been extensive recording of marine caves recently carried out in Italy [94], Corsica [95], Croatia [96], and Greece (especially surveys on *M. monachus* cave shelters [97]). The results of the present study provide information on the extensive distribution of *P. oceanica* meadows in some eastern Mediterranean countries that was lacking until recently (see [50]), particularly Turkey, Greece, Cyprus, and Croatia. Although very limited data were found for the presence of coralligenous formations in the southern and eastern coasts of the Levantine Sea, the current work reveals an extensive presence of this habitat in the poorly studied Adriatic, Aegean and Ionian Seas, and the northern Levantine coasts. This finding substantially contributes to the improvement of the previously existing knowledge gap regarding their presence in these regions, as acknowledged by [98], although further mapping is required to determine the full extent of this highly variable habitat. Furthermore, we provide the first map on the distribution of marine caves in the Mediterranean Sea. However, detailed censuses in each country are still needed to fill current distribution gaps, while further underwater studies in the eastern and southern countries (e.g., Greece, Turkey, and Libya) are expected to significantly raise the number of caves in the corresponding ecoregions. The high level of individuality [99] and fragmentation [41], [100] that characterizes marine cave assemblages emphasizes this need.

Regardless of our efforts to obtain information on the distribution of the selected habitats from southern Mediterranean countries (particularly Egypt, Libya, and Algeria), we did not manage to collect a substantial amount of data as they were either lacking or, when available, rigid bureaucratic structures made access to them almost impossible. The establishment of long-term relationships with research institutions and governmental agencies in these countries may facilitate data availability. Species distribution and habitat suitability models could assist with filling the gaps in data poor regions [7], in which case we suggest the use of MarProb (Marxan with probabilities) to accommodate mapping accuracy and account for uncertainty related to the biodiversity spatial distribution [101]. Ideally, coverage data should replace presence/absence data, allowing for quantitative instead of qualitative assessments of what is already protected in the Mediterranean and what is not (see results). Moreover, outstanding marine caves and coralligenous assemblages with unique species composition and high levels of endemism should be regarded as distinct conservation features. The health of *P. oceanica* meadows should also be taken into account in future prioritization analyses, as presence/absence or even coverage data alone cannot guarantee the persistence of the meadow [102]. Rather than a complete representation of the distribution of the examined habitats, our work proposes a methodology that addresses data heterogeneity and ecoregional representation in large-scale conservation planning. This approach should be repeated and our results modified as new data on their distribution become available. Issues of connectivity and minimum size of protected area should also be incorporated into this systematic approach, when relevant information becomes available, in order to ensure gene flow and persistence of populations [see 103]. Furthermore, future work should incorporate the distribution of more habitat types of conservation interest listed in detailed classification systems such as EUNIS [104].

The uneven distribution of data, especially of coralligenous formations and marine caves, is not only a matter of invested research effort or data availability, but also depends on the geomorphological heterogeneity of the Mediterranean coastline and seabed; the northern basin encompasses 92.3% of the Mediterranean rocky coastline, while south and extreme south-eastern areas are dominated by sandy coasts [49]. Hence, the concentration of a high number of marine cave systems and the extensive distribution of coralligenous in the Adriatic, Aegean, and Tyrrhenian Seas is highly related to the presence of extensive rocky coasts in these areas, with Italy, Greece, and Croatia covering 74% of the Mediterranean's rocky coasts [49]. As for *P. oceanica*, it is worth noting the reported absence of its meadows in the extreme south-east of the basin (specifically in the eastern coast of Turkey, the Syrian and Lebanese coasts, and the eastern coast of Egypt), in the northern Italian coast of the Adriatic Sea, and the south-western extreme of the Alboran Sea, due to unfavorable temperature or salinity conditions related to river discharge (namely Nile and Po Rivers) [105]–[107].

Bode et al. [108] found that the identification of priority areas is more sensitive to the inclusion of cost data than biodiversity data, highlighting the necessity to consider both ecological and economic data in prioritization schemes. In this context, our systematic approach for the identification of priority areas accounted for fisheries and aquaculture opportunity cost. Such applications are scarce in the Mediterranean Sea at a local scale [60], [61], [109], [110] and until now non-existent at a basin-scale [7]. While overlaying species distributions and spatial patterns of human threats to identify hotspots is scientifically interesting, such exercises are of limited practical use for conservation (Fraschetti et al. unpublished data). Taking action in areas of conflict between conservation and other human activities may not be feasible from a social or economic point of view. On the other hand, identifying priorities for conservation in locations where the targets for the conservation features are met while opportunity cost for conflicting social groups (in our case fishers and fish farmers) is more equitably allocated, is likely to lead to more viable solutions. Acknowledging the limitations of our methods for the cost estimation, we encourage their improvement as soon as additional data become available, through the process of adaptive management [111]. This could be done by incorporating illegal, unregulated, and unreported fishing into the GFCM data, and by using information from Vessel Monitoring Systems applied in large-scale fisheries in most Mediterranean countries for better estimation of their spatial distribution [109], [110]. Furthermore, social aspects of artisanal fisheries should be taken into account. Although lower opportunity cost was observed towards the north-eastern Mediterranean areas, and especially the insular areas of the Adriatic and Aegean Sea, it should be noted that artisanal fisheries constitute an important sector of primary production, despite its small contribution to the annual Gross National Product, as it facilitates social and economic cohesion by creating job opportunities and income security that are especially important for rural areas and remote islands (e.g., Aegean Islands [112]). Future systematic plans should also include socioeconomic data related to other important economic sectors in the region such as tourism (see [61], [110]).

Spatial overlap between current Mediterranean MPAs and priority areas identified at the ecoregional planning scale was higher in the northwestern Mediterranean Sea (i.e. the Algero-Provencal Basin and Tyrrhenian Sea) as well as in the Ionian Sea, especially due to the presence of Natura 2000 sites in these ecoregions. Croatia's entry into the EU in July 2013 and its subsequent proposal of Natura 2000 sites is expected to significantly increase the overlap between MPAs and priority areas in the Adriatic Sea (one of the two ecoregions with the lowest overlap). It is worth mentioning that in the present analysis only MPAs belonging to Croatian national categories were taken into account. The mismatch between our priority areas and current MPAs in the Algero-Provencal Basin and Tyrrhenian Sea is justified by the presence of large off-shore protected areas, SPAMIs, mainly targeting conservation of pelagic features (e.g., marine mammals within the Pelagos Sanctuary). Gabrie et al. [46] found that *P. oceanica* meadows are fairly well represented in the western Mediterranean basin since 49.7% of their distribution is found within the limits of MPAs (aside Pelagos sanctuary), out of which 19.1% is found within MPAs that have a management structure, while coralligenous habitats are represented at a level of 11.6% of all MPAs (aside Pelagos sanctuary), out of which 4.9% is in MPAs that have a management structure. On the other hand, habitat representation in the eastern Mediterranean Sea is considered poor; despite the presence of several marine Natura 2000 sites most of them currently have no established management structure. Furthermore, a considerable proportion of eastern Mediterranean MPAs is poorly studied, species' and habitats' inventories remain incomplete and few management plans or monitoring schemes are being implemented [46]. Therefore, before the creation of new MPAs or the enlargement of current MPAs, the management adequacy and viability of those MPAs should be reinforced through enhanced resources, such as long-term monitoring programs, capacity building programs, connectivity studies, and the creation of sustainable financial instruments to ensure enforcement of MPAs [46].

However, MPAs alone cannot safeguard the conservation of *P. oceanica* meadows, coralligenous formations and cave habitats [21], [113]. In urban coastal areas, the establishment of sewage treatment plants is an additional conservation action to be considered in order to reduce pollution and water turbidity [12], [114]. Furthermore, damage due to coastal development could be reduced by: a) setting minimum distances between artificial rip-rap (i.e. ports, breakwaters, areas reclaimed from the sea) and key habitats, b) using geotextile screens around the building sites to minimize turbidity caused, and c) avoiding any construction work during summer when *P. oceanica* is reconstituting its reserves [12]. Damage from anchoring should also be controlled by the delineation of fix mooring points and the establishment of ecological moorings where *P. oceanica* meadows and coralligenous habitats occur [115]. Mitigation of the mechanical damage to coralligenous formations caused by recreational fisheries could be achieved through the exclusion of long-lines and nets from areas with dense populations of erect invertebrates [116], [117]. Furthermore, as coralligenous assemblages and marine caves are a pole of attraction for SCUBA divers, the conservation of these habitats requires specific regulations regarding the number and experience level of divers [45], [118]. Implementation of quotas on the maximum number of divers, pre-dive briefings, and awareness raising campaigns can be effective measures to reduce destruction in certain locations [119]–[121]. Nevertheless, a prerequisite to quantification of threats and effective implementation of conservation actions is the acquirement of fine scale spatial data, especially for coralligenous habitats and marine caves, which are less studied than *P. oceanica* meadows. Information on depth distribution and present ecological status of coralligenous habitats and marine cave assemblages is urgently needed [21], [41].

The present study, despite its limitations, builds upon previous efforts focusing on large-scale spatial prioritization in the Mediterranean Sea, e.g., [7], [48]. As opposed to most previous schemes (see [7] and references therein) our proposal a) is based on the distribution of habitats and not on the distribution of species, b) explicitly considers the spatial variability of cost and rejects the false assumption that cost for conservation is the same throughout this socially, economically and politically heterogeneous basin, and c) suggests that planning at ecoregional scale is the largest appropriate scale for identifying priority areas in which adequate conservation of biodiversity (ecosystems, species, genes) will be achieved.

## Supporting Information

Text S1 (DOC)

## References

[1] Lourie SA, Vincent ACJ (2004) Using biogeography to help set priorities in marine conservation. Conservation Biology 18: 1004–1020.

[2] Dorries MB, Van Dover CL (2003) Higher-taxon richness as a surrogate for species richness in chemosynthetic communities. Deep-Sea Research I 50: 749–755.

[3] McArthur MA, Brooke BP, Przeslawski R, Rayan DA, Lucieer VL, et al (2010) On the use of abiotic surrogates to describe marine benthic biodiversity. Estuarine, Coastal and Shelf Science 88: 21–32.

[4] Ward TJ, Vanderklift MA, Nicholls AO, Kenchington RA (1999) Selecting marine reserves using habitats and species assemblages as surrogates for biological diversity. Ecological Applications 9: 691–698.

[5] Beck MW, Odaya M (2001) Ecoregional planning in marine environments: identifying priority sites for conservation in the northern Gulf of Mexico. Aquatic Conservation: Marine and Freshwater Ecosystems 11: 235–242.

[6] Fernandes L, Day J, Lewis A, Slegers S, Kerrigan B, et al (2005) Establishing representative no-take areas in the Great Barrier Reef: Large-scale implementation of theory on marine protected areas. Conservation Biology 19: 1733–1744.

[7] Micheli F, Levin N, Giakoumi S, Katsanevakis S, Abdulla A, et al (2013) Setting priorities for regional conservation planning in the Mediterranean. PLoS One 8: e59038. 23577060 10.1371/journal.pone.0059038 PMC3618442

[8] Andelman SJ, Fagan WF (2000) Umbrellas and flagships: Efficient conservation surrogates or expensive mistakes?. Proceedings of the National Academy of Sciences of the United States of America 97: 5954–5959. 10811901 10.1073/pnas.100126797 PMC18540

[9] SPA/BD (1995) Protocol concerning Specially Protected Areas and Biological Diversity in the Mediterranean. Barcelona Convention, Barcelona, 9–10 June 1995.

[10] Fraschetti S, Terlizzi A, Boero F (2008) How many habitats are there in the sea (and where)? Journal of Experimental Marin Biology & Ecology 366: 109–115.

[11] Francour P (1997) Fish assemblages of Posidonia oceanica beds at Port-Cros (France, NW Mediterranean): assessment of composition and long-term fluctuations by visual census. Marine Ecology 18: 157–173.

[12] Boudouresque CF, Bernard G, Bonhomme P, Charbonnel E, Diviacco G, et al.. (2012) Protection and conservation of Posidonia oceanica meadows. Tunis: RAMOGE and RAC/SPA. 1–202 p.

[13] Liquete C, Piroddi C, Drakou EG, Gurney L, Katsanevakis S, et al (2013) Current status and future prospects for the assessment of marine and coastal ecosystem services: a systematic review. PLoS One 8 (7): e67737. 10.1371/journal.pone.0067737 PMC3701056 23844080

[14] Waycott M, Duarte CM, Carruthers TJB, Orth RJ, Dennison WC, et al (2009) Accelerating loss of seagrasses across the globe threatens coastal ecosystems. Proceedings of the National Academy of Sciences of the United States of America 106: 12377–12381. 19587236 10.1073/pnas.0905620106 PMC2707273

[15] Bonacorsi M, Pergent-Martini C, Breand N, Pergent M (2013) Is *Posidonia oceanica* regression a general feature in the Mediterranean Sea? Mediterranean Marine Science 14: 193–203.

[16] Boudouresque CF, Guillaume B, Pergent G (2009) Regression of Mediterranean seagrasses caused by natural processes and anthropogenic disturbances and stress: a critical review. Botanica Marina 52: 395–418.

[17] Jorda G, Marba N, Duarte CM (2012) Mediterranean seagrass vulnerable to regional climate change. Nature Climate Change 2: 821–824.

[18] Marba N, Duarte CM, Holmer M, Martinez R, Basterretxea G, et al (2002) Effectiveness of protection of seagrass (*Posidonia oceanica*) populations in Cabrera National Park (Spain). Environmental Conservation 29: 509–518.

[19] Boudouresque CF (2004) Marine biodiversity in the Mediterranean: status of species, populations and communities. Scientific Reports of Port-Cros National Park 20: 97–146.

[20] UNEP-MAP-RAC/SPA (2008) Action plan for the conservation of the coralligenous and other calcareous bio-concretions in the Mediterranean Sea. Tunis: Ed. RAC/SPA. 1–21 p.

[21] Ballesteros E (2006) Mediterranean coralligenous assemblages: A synthesis of present knowledge. Oceanography and Marine Biology: An Annual Review 44: 123–195.

[22] Bianchi CN, Morri C (2000) Marine biodiversity of the Mediterranean Sea: Situation, problems and prospects for future research. Marine Pollution Bulletin 40: 367–376.

[23] Coll M, Piroddi C, Kaschner K, Ben Rais Lasram F, Steenbeek J, et al (2010) The Biodiversity of the Mediterranean Sea: Estimates, Patterns, and Threats. PLoS One 5: e11842. 20689844 10.1371/journal.pone.0011842 PMC2914016

[24] Gili JM, Coma R (1998) Benthic suspension feeders: their paramount role in littoral marine food webs. Trends in Ecology and Evolution 13: 316–321. 21238320 10.1016/s0169-5347(98)01365-2

[25] Sartoretto S, Francour P (1997) Quantification of bioerosion by Sphaerechinus granularis on “coralligène” concrections of the western Mediterranean. Journal of Marine Biological Association of United Kingdom 77: 565–568.

[26] Garrabou J, Ballesteros E (2000) Growth of *Mesophyllum alternans* and *Lithophyllum frondosum* (Corallinales, Rhodophyta) in the northwestern Mediterranean. European Journal of Phycology 35: 1–10.

[27] Cerrano C, Bavestrello G, Bianchi CN, Calcinai B, Cattaneo-Vietti R, et al.. (2001) The role of sponge bioerosion in Mediterranean coralligenous accretion. In: Faranda FM, Guglielmo L, Spezie G, editors. Mediterranean Ecosystems: Structures and Processes. 235–240.

[28] Teixidó N, Garrabou J, Harmelin JG (2011) Low dynamics, high longevity and persistence of sessile structural species dwelling on Mediterranean coralligenous outcrops. PLoS One 6: e23744–e23744. 21887308 10.1371/journal.pone.0023744 PMC3161055

[29] Cebrian E, Linares C, Marschal C, Garrabou J (2012) Exploring the effects of invasive algae on the persistence of gorgonian populations. Biological Invasions 14: 2647–2656.

[30] Piazzi L, Balata D, Ceccherelli G, Cinelli F (2005) Interactive effect of sedimentation and *Caulerpa racemosa var. cylindracea* invasion on macroalgal assemblages in the Mediterranean Sea. Estuarine, Coastal and Shelf Science 64: 467–474.

[31] Piazzi L, Gennaro P, Balata D (2012) Threats to macroalgal coralligenous assemblages in the Mediterranean Sea. Marine Pollution Bulletin 64: 2623–2629. 22863350 10.1016/j.marpolbul.2012.07.027

[32] Teixidó N, Casas E, Cebrián E, Linares C, Garrabou J (2013) Impacts on Coralligenous Outcrop Biodiversity of a Dramatic Coastal Storm. PLoS One 8: e53742. 23326496 10.1371/journal.pone.0053742 PMC3542355

[33] Ballesteros E (2003) The coralligenous in the Mediterranean Sea: Definition of the coralligenous assemblage in the Mediterranean, its main builders, its richness and key role in benthic ecology as well as its threats. Project for the preparation of a Strategic Action Plan for the Conservation of the Biodiversity in the Mediterranean Region (SAP BIO). UNEP-MAP-RAC/SPA. 1–87 p.

[34] UNEP-MAP-RAC/SPA (In press) Propositions pour la mise en place d'un plan d'action relatif à la conservation des peuplements obscurs de Méditerranée. MarseilleFrance: UNEP-MAP-RAC/SPA. 1–16 p.

[35] Riedl R (1966) Biologie der Meereshöhlen. HamburgGermany: Paul Parey. 1–636 p.

[36] Vacelet J, Boury-Esnault N, Harmelin JG (1994) Hexactinellid cave, a unique deep-sea habitat in the scuba zone. Deep-Sea Research I 41: 965–973.

[37] Harmelin JG, Vacelet J (1997) Clues to deep-sea biodiversity in a nearshore cave. Vie Milieu 47: 351–354.

[38] Dendrinos P, Karamanlidis AA, Kotomatas S, Legakis A, Tounta E, et al (2007) Pupping habitat use in the Mediterranean monk seal: a long-term study. Marine Mammal Science 23: 615–628.

[39] Harmelin JG, Vacelet J, Vasseur P (1985) Les grottes sous-marines obscures: un milieu extrême et un remarquable biotope refuge. Téthys 11: 214–229.

[40] Muricy G, Solé-Cava AM, Thorpe JP, Boury Esnault N (1996) Genetic evidence for extensive cryptic speciation in the subtidal sponge *Plakina trilopha* (Porifera: Demospongiae: Homoscleromorpha) from the Western Mediterranean. MArine Ecology Progress Series 138: 181–187.

[41] Gerovasileiou V, Voultsiadou E (2012) Marine Caves of the Mediterranean Sea: A Sponge Biodiversity Reservoir within a Biodiversity Hotspot. PLoS One 7: e39873. 22808070 10.1371/journal.pone.0039873 PMC3394755

[42] Chevaldonné P, Lejeusne C (2003) Regional warming-induced species shift in north-west Mediterranean marine caves. Ecological Letters 6: 371–379.

[43] Bussoletti E, Cottingham D, Bruckner A, Roberts G, Sandulli R (2010) Proceedings of the International Workshop on Red Coral Science, Management, and Trade: Lessons from the Mediterranean. Silver Spring, U.S.A. 1–233.

[44] Parravicini V, Guidetti P, Morri C, Montefalcone M, Donato M, et al (2010) Consequences of sea water temperature anomalies on a Mediterranean submarine cave ecosystem. Estuarine Coastal and Shelf Science 86: 276–282.

[45] Guarnieri G, Terlizzi A, Bevilacqua S, Fraschetti S (2012) Increasing heterogeneity of sensitive assemblages as a consequence of human impact in submarine caves. Marine Biology 159: 1155–1164.

[46] Gabrié C, Lagabrielle E, Bissery C, Crochelet E, Meola B, et al.. (2012) Statut des Aires Marines Protégées en mer Méditerranée. MedPAN & CAR/ASP. 1–260 p.

[47] Coll M, Piroddi C, Albouy C, Ben Rais Lasram F, Cheung WWL, et al (2012) The Mediterranean under siege: spatial overlap between marine biodiversity, cumulative threats and marine reserves. Global Ecology and Biogeography 21: 465–481.

[48] Giakoumi S, Mazor T, Fraschetti S, Kark S, Portman M, et al (2012) Advancing marine conservation planning in the Mediterranean Sea. Reviews in Fish Biology and Fisheries 22: 943–949.

[49] Stewart IS, Morhange C (2009) Coastal geomorphology and sea-level change. In: Woodward JC, editor. Oxford, U.K.: Oxford University Press. 385–413.

[50] Abdulla A, Gomei M, Maison E, Piante C (2008) Status of Marine Protected Areas in the Mediterranean Sea. Malaga and France: IUCN and WWF. 1–152 p.

[51] Spalding MD, Fox HE, Allen GR, Davidson N, Ferdana ZA, et al (2007) Marine ecoregions of the world: A bioregionalization of coastal and shelf areas. BioScience 57: 573–583.

[52] Spalding M, Agostini V, Grant S, Rice J (2012) Pelagic provinces of the world: a biogeographic classification of the world's surface pelagic waters. Ocean and Coastal Management 90: 19–30.

[53] Watling L, Guinotte JM, Clark M, Smith C (2013) A proposed biogeography of the deep ocean floor. Progress in Oceanography 111: 91–112.

[54] Mouillot D, Albouy C, Guilhaumon F, Ben Rais Lasram F, Coll M, et al (2011) Protected and threatened components of fish biodiversity in the Mediterranean Sea. Current Biology 21: 1044–1050. 21658949 10.1016/j.cub.2011.05.005

[55] Moranta J, Palmer M, Morey G, Ruiz A, Morales-Nin B (2006) Multi-scale spatial variability in fish assemblages associated with Posidonia oceanica meadows in the Western Mediterranean Sea. Estuarine, Coastal and Shelf Science 68: 579–592.

[56] Kalogirou S, Corsini Foka M, Sioulas A, Wennhage H, Pihl L (2010) Diversity, structure and function of fish assemblages associated with Posidonia oceanica beds in an area of the eastern Mediterranean Sea and the role of non-indigenous species. Journal of Fish Biology 77: 2338–2357. 21155787 10.1111/j.1095-8649.2010.02817.x

[57] Abbiati M, Costantini F, Fauvelot C (2009) Conservation of coralligenous reefs: effective larval dispersal, scales of connectivity and resilience. In: Pergent-Martini C, Brichet M, editors. Proceedings of the 1st symposium on conservation of the coralligenous bio-concretions. Tabarka: RAC/SPA Publications. 269.

[58] Mokhtar-Jamaï K, Pascual M, Ledoux J-B, Coma R, Féral P, et al (2011) From global to local genetic structuring in the red gorgonian Paramuricea clavata: the interplay between oceanographic conditions and limited larval dispersal. Molecular Ecology 20: 3291–3305. 21762434 10.1111/j.1365-294X.2011.05176.x

[59] Abdulla A, Gomei M, Hyrenbach D, Notarbartolo-di-Sciara G, Agardy T (2009) Challenges facing a network of representative marine protected areas in the Mediterranean: prioritizing the protection of underrepresented habitats. ICES Journal of Marine Science 66: 22–28.

[60] Fraschetti S, D'Ambrosio P, Micheli F, Pizzolante F, Bussotti S, et al (2009) Design of marine protected areas in a human-dominated seascape. Marine Ecology Progress Series 375: 13–24.

[61] Giakoumi S, Grantham HS, Kokkoris GD, Possingham HP (2011) Designing a network of marine reserves in the Mediterranean Sea with limited socio-economic data. Biological Conservation 144: 753–763.

[62] Naidoo R, Balmford A, Ferraro PJ, Polasky S, Ricketts TH, et al (2006) Integrating economic costs into conservation planning. Trends in Ecology & Evolution 21: 681–687. 17050033 10.1016/j.tree.2006.10.003

[63] Ban NC, Hansen GJA, Jones M, Vincent ACJ (2009) Systematic marine conservation planning in data-poor regions: Socioeconomic data is essential. Marine Policy 33: 794–800.

[64] Richardson EA, Kaiser MJ, Edwards-Jones G, Possingham HP (2006) Sensitivity of marine-reserve design to the spatial resolution of socioeconomic data. Conservation Biology 20: 1191–1202. 16922235 10.1111/j.1523-1739.2006.00426.x

[65] Ando A, Camm J, Polasky S, Solow A (1998) Species distributions, land values and efficient conservation. Science 279: 2126–2128. 9516117 10.1126/science.279.5359.2126

[66] Pauly D, Christensen V, Guenette S, Pitcher TJ, Sumaila UR, et al (2002) Towards sustainability in world fisheries. Nature 418: 689–695. 12167876 10.1038/nature01017

[67] Notarbartolo di Sciara G, Agardy T (2010) Overview of scientific findings and criteria relevant to identifying SPAMIs in the Mediterranean open seas, including the deep sea. Tunis: UNEP-MAP. 1–71 p.

[68] Game ET, Kareiva P, Possingham HP (2013) Six common mistakes in conservation priority setting. Conservation Biology 27: 480–485. 23565990 10.1111/cobi.12051 PMC3732384

[69] Portman ME, Notarbartolo di Sciara G, Agardy T, Katsanevakis S, Possingham HP, et al (2013) He who hesitates is lost: Why conservation in the Mediterranean Sea is necessary and possible now. Marine Policy 42: 270–279.

[70] Annoni A, Luzet C, Gubler E, Ihde J (2001) Map projections for Europe, EUR 20120. European Commission.

[71] Mazor T, Possingham HP, Kark S (2013) Collaboration among countries in marine conservation can achieve substantial efficiencies. Diversity and Distributions: doi: 10.1111/ddi 12095.

[72] FAO (2011) GFCM Task 1 Statistical Bulletin 2008, Food and Agriculture Organisation of the United Nations, General fisheries commission for the Mediterranean. Available: http://www.gfcm.org/gfcm/topic/17106/en. Accessed 2012 Dec 30.

[73] FAO (2010) Globefish European Price Report. Italy, Rome: Food and Agriculture Organization of the United Nations, Fish Products and Industry Division, Italy, Rome.

[74] Lleonart J, Maynou F (2003) Fish stock assessments in the Mediterranean: state of the art. Scientia Marina 67: 37–49.

[75] European Commission (2008) Eurostat statistics in focus, Agriculture and fisheries. Available: http://ec.europa.eu/eurostat. Accessed 2013 May 15.

[76] Lloret J, Font T (2013) A comparative analysis between recreational and artisanal fisheries in a Mediterranean coastal area. Fisheries Management and Ecology 20: 148–160.

[77] Gaudin C, De Young C (2007) Recreational fisheries in the Mediterranean countries: a review of existing legal frameworks. Studies and Reviews. RomeItaly: General Fisheries Commission for the Mediterranean, FAO. 1–85 p.

[78] Ünal V, Acarli D, Gordoa A (2010) Characteristics of Marine Recreational Fishing in the Çanakkale Strait (Turkey) Mediterranean. Marine Science 11: 315–330.

[79] ICES CM (2008) First results from a pilot survey of recreational fishing in France; 22–26 September. 2008: 15.

[80] Trujillo P, Piroddi C, Jacquet J (2012) Fish farms at sea: The ground truth from Google Earth. PLoS One 7: e30546. 22347383 10.1371/journal.pone.0030546 PMC3275594

[81] Ball IR, Possingham HP, Watts M (2009) Marxan and relatives: Software for spatial conservation prioritisation. In: Moilanen A, Wilson KA, Possingham HP, editors. Spatial conservation prioritisation: Quantitative methods and computational tools. Oxford, UK: Oxford University Press. 185–195.

[82] European Topic Centre on Biological Diversity (ETC/BD) (2010). Available: http://bd.eionet.europa.eu/activities/Natura_2000/pdfs/Additional_marine_guidelines.pdf.Accessed 2013 Feb 20.

[83] Stewart RR, Possingham HP (2005) Efficiency, costs and trade-offs in marine reserve system design. Environmental Modeling & Assessment 10: 203–213.

[84] MAPAMED (2013) The database on Mediterranean Marine Protected Areas. MedPAN, RAC/SPA. Available: www.mapamed.org. Accessed 2013 May 15.

[85] Morales-Nin B, Moranta J, García C, Tugores MP, Grau AM, et al (2005) The recreational fishery off Majorca Island (western Mediterranean): some implications for coastal resource management. ICES Journal of Marine Science 62: 727–739.

[86] Kark S, Levin N, Grantham H, Possingham HP (2009) Between-country collaboration and consideration of costs increase conservation planning efficiency in the Mediterranean Basin. Proceedings of the National Academy of Sciences of the United States of America 106: 15368–15373. 19717457 10.1073/pnas.0901001106 PMC2741257

[87] Barr LM, Possingham HP (2013) Are outcomes matching policy commitments in Australian marine conservation planning? Marine Policy 42: 39–48.

[88] Voultsiadou E (2009) Reevaluating sponge diversity and distribution in the Mediterranean Sea. Hydrobiologia 628: 1–12.

[89] Lejeusne C, Chevaldonne P, Pergent-Martini C, Boudouresque CF, Perez T (2010) Climate change effects on a miniature ocean: the highly diverse, highly impacted Mediterranean Sea. Trends in Ecology & Evolution 25: 250–260. 19959253 10.1016/j.tree.2009.10.009

[90] Zenetos A, Gofas S, Morri C, Rosso A, Violanti D, et al (2012) A contribution to the application of European Union's marine strategy framework directive (MSFD). Part 2. Introduction trends and pathways. Mediterranean Marine Science 13: 328–352.

[91] Halpern BS, Klein CJ, Brown CJ, Beger M, Grantham HS, et al (2013) Achieving the triple bottom line in the face of inherent trade-offs among social equity, economic return, and conservation. Proceedings of the National Academy of Sciences of the United States of America 110: 6229–6234. 23530207 10.1073/pnas.1217689110 PMC3625307

[92] OCEANA (2008) Estudio bionómico de Cabrera: Estudio bionómico de los fondos profundos del Parque Nacional Maritimo Terrestre del Archipiélago de Cabrera y sus Alrededores. OCEANA y Govern de les Illes Balears. 1–60 p.

[93] Bianchi CN, Morri C, Navone A (2010) I popolamenti delle scogliere rocciose sommerse dell'Area Marina Protetta di Tavolara Punta Coda Cavallo (Sardegna nord-orientale). Travaux scientifiques du Parc national de Port-Cros 24: 39–85.

[94] Cicogna F, Bianchi CN, Ferrari G, Forti P (2003) Le grotte marine: cinquant‘anni di ricerca in Italia. RomeItaly: Ministero dell’Ambiente e della Tutela del Territorio. 1–505 p.

[95] CREOCEAN-DREAL (2010) Recensement des grottes submergées ou semi-submergées sur le littoral Corse. 1–80 p.

[96] Surić M, Lončarić R, Lončar N (2010) Submerged caves of Croatia: distribution, classification and origin. Environmental Earth Sciences 61: 1473–1480.

[97] MOm (2009) Annual Technical Report 2008, on the Status of the Mediterranean Monk Seal (*Monachus monachus*) in Greece. Athens, Greece. 1–15 p.

[98] UNEP-MAP-RAC/SPA (2009) State of knowledge of the geographical distribution of the coralligenous and other calcareous bio-concretions in the Mediterranean. UNEP (DEPI)/MED WG. 331/Inf.6. 1–167 p.

[99] Harmelin JG (1985) Organisation spatiale des communautés sessiles des grottes sous-marines de Méditerranée. Rapp Comm Int Mer Médit 29: 149–153.

[100] Lejeusne C, Chevaldonné P (2006) Brooding crustaceans in a highly fragmented habitat: the genetic structure of Mediterranean marine cave-dwelling mysid populations. Molecular Ecology 15: 4123–4140. 17054507 10.1111/j.1365-294X.2006.03101.x

[101] Tulloch VJ, Possingham HP, Jupiter SD, Roelfsema C, Tulloch AI, et al. (In Press) Incorporating uncertainty associated with habitat data in marine reserve design. Biological Conservation 162: 41–51.

[102] Montefalcone M, Albertelli G, Morri C, Bianchi CN (2007) Urban seagrass: status of Posidonia oceanica off Genoa city waterfront (Italy). Marine Pollution Bulletin 54: 206–213. 17113606 10.1016/j.marpolbul.2006.10.005

[103] Beger M, Linke S, Watts M, Game E, Treml E, Ball I, Possingham HP (2010) Incorporating asymmetric connectivity into spatial decision making for conservation. Conservation Letters 3: 359–368.

[104] Salomidi M, Katsanevakis S, Borja Á, Braeckman U, Damalas D, et al (2012) Assessment of goods and services, vulnerability, and conservation status of European seabed biotopes: a stepping stone towards ecosystem-based marine spatial management. Mediterranean Marine Science 13: 49–88.

[105] Celebi B, Gucu AC, Ok M, Sakinan S, Akoglu E (2006) Hydrographic indications to understand the absence of Posidonia oceanic in the Levant sea (Eastern Mediterranean). Biologia Marina Mediterranea 13: 34–38.

[106] Romero J, Martínez-Crego B, Alcoverro T, Pérez M (2007) A multivariate index based on the seagrass Posidonia oceanica (POMI) to assess ecological status of coastal waters under the water framework directive (WFD). Marine Pollution Bulletin 55: 196–204. 17045301 10.1016/j.marpolbul.2006.08.032

[107] Pergent G, Bazairi H, Bianchi CN, Boudouresque CF, Buia MC, et al.. (2012) Mediterranean Seagrass Meadows: Resilience and Contribution to Climate Change Mitigation, A Short Summary. GlandSwitzerland and MálagaSpain: IUCN. 1–40 p.

[108] Bode M, Wilson K, Brooks T, Turner W, McBride MT, et al (2008) Cost-effective global conservation spending is robust to taxonomic group. Proceedings of the National Academy of Sciences of the United States of America 105: 6498–6501. 18413614 10.1073/pnas.0710705105 PMC2359771

[109] Maiorano L, Bartolino V, Colloca F, Abella A, Belluscio A, et al (2009) Systematic conservation planning in the Mediterranean: a flexible tool for the identification of no-take marine protected areas. ICES Journal of Marine Science 66: 137–146.

[110] Giakoumi S, Katsanevakis S, Vassilopoulou V, Panayotidis P, Kavadas S, et al (2012) Could European marine conservation policy benefit from systematic conservation planning? Aquatic Conservation: Marine and Freshwater Ecosystems 22: 762–775.

[111] Katsanevakis S, Stelzenmüller V, South A, Sørensen TK, Jones PJS, et al (2011) Ecosystem-based marine spatial management: review of concepts, policies, tools, and critical issues. Ocean and Coastal Management 54: 807–820.

[112] Conides A (2007) Socio-economic status of the Hellenic capture fisheries sector. In: Papaconstantinou A, Zenetos A, Vassilopoulou V, Tserpes G, editors. State of Hellenic Fisheries. Athens: HCMR Publications. 172–178.

[113] Montefalcone M, Albertelli G, Morri C, Parravicini V, Bianchi CN (2009) Legal protection is not enough: Posidonia oceanica meadows in marine protected areas are not healthier than those in unprotected areas of the northwest Mediterranean Sea. Marine Pollution Bulletin 58: 515–519. 19150722 10.1016/j.marpolbul.2008.12.001

[114] Airoldi L (2003) The effects of sedimentation on rocky coastal assemblages. Oceanography and Marine Biology Annual Review 41: 161–203.

[115] Francour P, Magréau JF, Mannoni PA, Cottalorda JM, Gratiot J (2006) Management guide for Marine Protected Areas of the Mediterranean sea, Permanent Ecological Moorings. NiceFrance: Université de Nice-Sophia Antipolis & Parc National de Port-Cros. 1–68 p.

[116] Bavestrello G, Cerrano C, Zanzi D, Cattaneo-Vietti R (1997) Damage by fishing activities to the Gorgonian coral Paramuricea clavata in the Ligurian Sea. Aquatic Conservation: Marine Freshwater Ecosystems 7: 253–262.

[117] Maldonado M, López-Acosta M, Sánchez-Tocino L, Sitjà C (2013) The rare, giant gorgonian *Ellisella paraplexauroides*: demographics and conservation concerns. Marine Ecology Progress Series 479: 127–141.

[118] Rovere A, Parravicini V, Firpo M, Morri C, Bianchi CN (2011) Combining geomorphologic, biological and accessibility values for marine natural heritage evaluation and conservation. Aquatic Conservation: Marine and Freshwater Ecosystems 21: 541–552.

[119] Milazzo M, Chemello R, Badalamenti F, Camarda R, Riggio S (2002) The impact of human recreational activities in Marine Protected Areas: What lessons should be learnt in the Mediterranean Sea? Marine Ecology 23: 280–290.

[120] Medio D, Ormond RFG, Pearson M (1997) Effect of briefings on rates of damage to corals by SCUBA divers. Biological Conservation 79: 91–95.

[121] Luna B, Pérez V, Sánchez-Lizaso J (2009) Benthic impacts of recreational divers in a Mediterranean Marine Protected Area. ICES Journal of Marine Science 66: 517–523.

