# Supplementary material for: Ecoregion-Based Conservation Planning in the Mediterranean: Dealing with Large-Scale Heterogeneity
Source: PLoS One. 2013 Oct 14;8(10):e76449. doi: 10.1371/journal.pone.0076449 (PMC3796553; doi:10.1371/journal.pone.0076449)
Supplement: Text S1 — (DOC) [file pone.0076449.s001.doc]

**Sources of data on the distribution of *Posidonia oceanica* meadows**

Scientific and grey literature

Arnaud-Haond S, Migliaccio M, Diaz-Almela E, Texeira S, Van de Vliet MS, Alberto F, Procaccini G, Duarte CM, Serrao EA (2007) Vicariance patterns in the Mediterranean Sea: east-west cleavage and low dispersal in the endemic seagrass *Posidonia oceanica*. Journal of Biogeography 34, 963-976.

Basaram AK, Aksu M, Egemen O (2012) Impacts of the fish farms on the water column nutrient concentrations and accumulation of heavy metals in the sediments in the eastern Aegean Sea (Turkey). Environmental Monitoring and Assessment 162: 439-451

Belsher T, Houlgatte E, Boudouresque C-F (2005) Cartographie de la prairie a *Posidonia oceanica* et des principaux facies sédimentaires marins du Parc national de Port-Cros (Var, France, Méditerranée). Sci. Rep. Port-Cros natl. Park, Fr., 21: 19-28.

Ben Brahim M, Hamza A, Bouain A (2006) Distribution and time variability of micro-epithytes and phytoplankton in *Posidonia oceanica* meadows in the Kerkennah Islands (Tunisia). Biologia Marina Mediterranea 13 : 122-125.

Ben Brahim M, Hamza A, Bouain A (2006) Spatio-temporal distribution of macro-epiphytic organisms in *Posidonia oceanica* leaves in the Gulf of Gabes (Tunisia). Biologia Marina Mediterranea 13 : 126-129.

Ben Mustapha K, Afli A (2007) Quelques traits de la biodiversité marine de Tunisie: Proposition d’aires de conservation et de gestion. Report of the MedSudMed Expert Consultation on Marine Protected Areas and Fisheries Management. MedSudMed Technical Documents. Rome (Italy). pp. 32–55.

Ben Mustapha K, Komatsu T, Hattour A, Sammari Ch, Zarrouk S, et al. (2002) Tunisian mega benthos from infra (*Posidonia* meadows) and circalittoral (Coralligenous) sites. Bulletin de L’Institut National des Sciences et Techonlogies de Salammbô 29: 23–36.

Beqiraj S, Kashta L, Kuci M, Kasemi D, Mato X, Gace A (2008) Benthic macrofauna of *Posidonia oceanica* meadows in the Albanian coast. Natura Montenegrina, Podgorica, 7: 55-69.

Borg JA, Rowden AA, Attrill MJ, Schembri PJ, Jones MB (2009) Occurrence and distribution of different bed types of seagrass *Posidonia oceanica* around Maltese Islands. Mediterranean Marine Science10/2: 45-61

Bouiadjra BB, Taleb MZ, Marouf A, Benkada MY, Riadi H (2010) First record of the invasive alga *Caulerpa racemosa* (Caulerpales, Chlorophyta) in the Gulf of Arzew (western Algeria). Aquatic Invasions 5, Supplement 1: S97-101

Buskovic V, Macic V, Saveljic D, Ivanovic A (2004) Montenegro National Action Plan 1: Inventory and mapping of sensitive areas. In: National Action Plan (SAP BIO), UNEP-RAC/SPA and the Republic of Montenegro (Ministry of Environmental Protection and Physical Planning), Podgorica, Montenegro, 42 pp.

Calvo S, Tomasello A, Di Maida Di, Pirrotta M, Buia MC, Cinelli F, Cormaci M, Furnari G, Giaccone G, Luzzu F, Mazzola A, Orestano C, Procaccini G, Sara G, Scannavino A, Vizzini S (2010) Seagrasses along the Sicilian coasts. Chemistry and Ecology 26, Supplement: 249-266.

Cebrian J, Duarte CM, Marba N, Enriquez S, Gallegos M, Olesen B (1996) Herbivory on *Posidonia oceanica*: magnitude and variability in the Spanish Mediterranean. Marine Ecology Progress Series 130: 147-155.

Celebi B, Gucu AC, Ok M, Serdar S, Akoglu E (2007). Survival of the *Posidonia oceanica* transplanted into the northeastern Levant Sea. Rapp. Comm. Int. Mer. Médit., 38: 446.

Celebi B, Cemal Gucu A, Ok M, Sakinan S, Akoglu E (2006). Hydrographic indications to understand the absence of Posidonia oceanica in the Levant Sea (Eastern Mediterranean). Biol. Mar. Medit 13: 34-38.

Costantino G, Mastrototaro F, Tursi A, Torchia G, Pititto F, Salerno G, Lembo G, Sion L, D’Onghia G, Carlucci R, Maiorano P (2010) Distribution and bio-ecological features of *Posidonia oceanica* meadows along the coasts of the southern Adriatic and northern Ionian Seas. Chemistry and Ecology 26: 91-104.

Descamp P, Pergent G, Ballesta L, Foulquie M (2005) Underwater acoustic positioning systems as tool for *Posidonia oceanica* beds survey. C.R. Biologies 328: 75-80.

Djellouli A, Dupuy de la Grandrive R, Foulquié M, Al Mokhtar S (2008) Mission le long du littoral de Aïn El Ghazella (Libye). Programme « MedPosidonia » / CAR/ASP - Fondation d’entreprise TOTAL pour la Biodiversité et la Mer ; Mémorandum d’accord 01/CAR/ASP – MedPosidonia / 2007: 1-12.

Dolenec T, Lojen S, Lamba[sbreve]a S, Dolenec M (2006) Effects of fish farm loading on seagrass *Posidonia oceanica* at Vrgada Island (Central Adriatic): a nitrogen stable isotope study. Isotopes in Environmental and Health Studies 42: 77-85.

Elhaweet AE, Fishar MR, Geneid Y, Abdel-Moula E (2011) Assessment of fisheries and marine biodiversity of Sallum Gulf, Egypt. International Journal of Environmental Science and Engineering (IJESE) 1: 21-34.

Fernandez-Torquemada Y, Diaz-Valdes M, Colilla F, Luna B, Sanchez-Lizaso JL, Ramos-Espla AA (2008) Descriptors from *Posidonia oceanica* (L.) Delile meadows in coastal waters of Valencia, Spain, in the context of the EU Water Framework Directive. ICES Journal of Marine Science 65: 1492-1497.

Ferrario J (2011) Analisi dei dati sulle praterie a fanerogame marine (*Posidonia oceanica* e *Cymodocea nodosa*) nel tratto di costa tra Levanto e Monterosso. Master thesis, Universita degli Studi di Parma, 109pp.

Fourqurean JW, MarbaN, Duarte CM, Diaz-Almela E, Ruiz-Halpern S (2007) Spatial and temporal variation in the elemental and stable isotopic content of the seagrasses *Posidonia oceanica*  and *Cymodocea nodosa* from the Illes Balears, Spain. Marine Biology 151: 219-232.

Giakoumi S, Kokkoris GD (2013) Effects of habitat and substrate complexity on shallow sublittoral fish assemblages in the Cyclades Archipelago, North-eastern Mediterranean Sea. Mediterranean Marine Science 14, 58-68.

Guala I., Di Carlo G., Jakl Z., et al. (2012) Monitoring of *Posidonia oceanica* meadows in Croatian Protected Areas. Association Sunce Technical Report, January 2012, 40 pp. + Annexes.

Haddoud DA, Rawag A Marine Protected Areas along Libyan coast. [www.faomedsudmed.org/pdf/publications/TD3/TD3-Haddoud.pdf](http://www.faomedsudmed.org/pdf/publications/TD3/TD3-Haddoud.pdf)

Issaris Y, Katsanevakis S, Pantazi M, Vassilopoulou V, Panayotidis P, Kavadas S, et al. (2012) Ecological mapping and data quality assessment for the needs of ecosystem based marine spatial management: case study Greek Ionian Sea and the adjacent gulfs. Mediterranean Marine Science 13:297–311.

IUCN (2011) Towards a Representative Network of Marine Protected Areas in Libya. Gland, Switzerland and Málaga, Spain: IUCN. 68 pages.

Joksimovic D, Sankovic AR, Sankovic S (2011) Metal accumulation in a biological indicator (*Posidonia oceanica*) from the Montenegrin coast. Stud. Mar. 25: 37-58.

Kalogirou S, Corsini Foka M, Sioulas A, Wennhage H, Pihl L (2010) Diversity, structure and function of fish assemblages associated with *Posidonia oceanica* beds in an area of the eastern Mediterranean Sea and the role of non-indigenous species. Journal of fish biology 77: 2338-2357.

Kocak F, Balduzzi A, Avni Benli H (2002) Epiphytic bryozoans community of *Posidonia oceanica* (L.) Delile meadow in the northern Cyprus (Eastern Mediterranean). Indian Journal of Marine Sciences 31: 235-238.

Koutsoubas D, Sini M, Evangelopoulos A, Batjakas I, Gerovasileiou V, Trygonis V, Poursanidis D, Georgakarakos S, Dounas C (2010) Investigation of the oceanographic and fisheries characteristics of the marine area of Psara Island – Preliminary Management Plan for the establishment of a Marine Protected Area. Final Report, Prefecture of Chios, Greece, 148 pp.

Kruzic P (2008) Variations in *Posidonia oceanica* meadow structure along the coast of the Dugi Otok Island (eastern Adriatic Sea) Journal of the Marine Biological Association of the United Kingdom 88: 883-892.

Leriche A, Boudouresque C-F, Monestiez P, Pasqualini V (2005) An improvement method to monitor the health of seagrass meadows based on kriging. Aquatic Botany 95: 51-54

Lopez y Royo C, Pergent G, Pergent-Martini C, Casazza G (2010) Seagrass (*Posidonia oceanica*) monitoring in western Mediterranean: implications for management and conservation. Environmental Monitoring and Assessment 171: 365-380.

Mayot N, Boudouresque C-F, Charbonnel E (2006) Changes over time of shoot density of the Mediterranean seagrass *Posidonia oceanica* at its depth limit. Biologia Marina Mediterranea 13: 250-254.

Mezali K, Zupo V, Francour P (2006) Population dynamics of *Holothuria (Holothuria) tubulosa* and *Holothuria (Lessonothuria) polii* of an Algerian *Posidonia oceanica* meadow. Biol. Mar. Medit 13: 158-161.

Meinesz A, Cirik S, Akcali B, Javel F, Migliaccio M, Thibaut T, Yuksek A, Procaccini G (2009) *Posidonia oceanica* in the Marmara Sea. Aquatic Botany 90: 18-22.

Milazzo M, Badalamenti F, Ceccherelli G, Chemello R (2004) Boat anchoring on *Posidonia oceanica* beds in a marine protected area (Italy, Western Mediterranean): effect of anchor types in different anchoring stages. Journal of Experimental Marine Biology and Ecology 299: 51–62.

Montefalcone M, Albertelli G, Morri C, Parravicini V, Bianchi CN (2009) Legal protection is not enough : *Posidonia oceanica* meadows in marine protected areas are not healthier than those in unprotected areas of the northwest Mediterranean Sea. Marine Pollution Bulletin 58: 515-519.

Montefalcone M, Chiantore M, Lanzone A, Morri C, Albertelli G, Bianchi CN (2008) BACI design reveals the decline of the seagrass *Posidonia oceanica* induced by anchoring. Marine Pollution Bulletin 56: 1637-1645.

Moranta J, Palmer M, Morey G, Ruiz A, Morales-Nin (2006) Multi-scale spatial variability in fish assemblages associated with Posidonia oceanica meadows in the Western Mediterranean Sea. Estuarine, Coastal and Shelf Science 68: 579-592.

Mostafa HM (2006). Preliminary ecological survey of seagrass beds at Marsa Matrouh, west of Alexandria-Egypt. Biologia Marina Mediterranea 13 : 72-76.

Okudan ES, Demir V, Kalkan E, Karhan SU (2011) Anchoring Damage on Seagrass- Meadows (Posidonia oceanica (L.) Delile) in Fethiye-Göcek Specially Protected Area (Eastern Mediterranean Sea, Turkey). In: Micallef, A. (ed.), MCRR3-2010 Conference Proceedings, Journal of Coastal Research,

Special Issue, No. 61, pp. 417-420. Grosseto, Tuscany, Italy, ISSN 0749-0208.

Pergent G, Djellouli A, Hamza A, Ettayeb K, Alkekli A, Talha M, Alkunti E (2006) Etude des communautés végétales benthiques dans les lagunes côtières d’Ain Al-Ghazala et de Farwa

(Libye). Centre d’Activité Régionale pour les Aires Spécialement Protégées : 64p + annexes.

Pergent-Martini C, Pasqualini V, Ferrat L, Pergent G (2006) Ecological data in integrated coastal zone management: case study of *Posidonia oceanica* meadows along the Corsican coast line (Mediterranean Sea). Environmental Management 38: 889-895.

Piazzi, Acunto S, Cinelli (2000) Mapping of *Posidonia oceanica* beds around Elba Island (western Mediterranean) with integration of direct and indirect methods. Oceanologica Acta 23: 339-346.

Pititto F, Ventrice A, Grenci S, Dedej Z, Kashta L, Beqiraj S, Gace A, Acunto S, Bulgheri G, Cinelli F,

Sivini N, Greco R, Torchia G (2009) Cartografia e protezione delle praterie di *Posidonia oceanica* lungo la costa Albanese. 40o Congresso della Societa Italian di Biologia Marina, Livorno (conference poster).

Planes S, Raventos N, Ferrari B, Alcoverro T (2011) Fish herbivory leads to shifts in seagrass *Posidonia oceanica* investments in sexual reproduction. Marine Ecology Progress Series 431: 205-213.

Romero J, Martinez-Crego B, Alcoverro T, Perez M (2007) A multivariate index based on the seagrass *Posidonia oceanica* (POMI) to assess ecological status of coastal waters under the water framework directive (WFD). Marine Pollution Bulletin 55: 196-204.

Sanchez-Jerez P, Barbera-Cebrian C, Ramos-Espla AA (2000) Influence of the structure of *Posidonia oceanica* meadows modified by bottom trawling on crustacean assemblages: comparison of amphipods and decapods. Scientia Marina 64: 319-326.

Sghaier YR, Zakhama-Sraieb R, Charfi-Cheikhrouha F (2013) Patterns of shallow seagrass (*Posidonia oceanica*) growth and flowering along the Tunisian coast. Aquatic Botany 104: 185-192.

Simeone S (2008) *Posidonia oceanica* banquettes removal: sedimentological, geomorphological and ecological implilcations. PhD thesis, Universita degli studi della Tuscia, pp 127.

SoHelME (2005) State of the Hellenic Marine Environment. E. Papathanassiou & Zenetos, A., HCMR, 360pp.

Soualili D, Guillou M (2009) Variation in the reproductive cycle of the sea urchin *Paracentrotus lividus* in three differently polluted locations near Algiers (Algeria). Marine Biodiversity Records 2, DOI: <http://dx.doi.org/10.1017/S175526720900092X>

Tomas F, Turon X, Romero J (2005) Seasonal and small-scale spatial variability of herbivory pressure on the temperate seagrass *Posidonia oceanica*. Marine Ecology Progress Series 301: 95-107.

UNEP-MAP RAC/SPA (2009) State of knowledge on the geographical distribution of marine magnoliophyta meadows in the Mediterranean. By Leonardini R, Pergent G, Boudouresque C-F (eds). RAC/SPA, Tunis, pp. 374.

UNEP-MAP-RAC/SPA (2007) Proceedings of the third Mediterranean symposium on marine vegetation (Marseilles, 27-29 March 2007). C Pergent-Martini, S El Asmi, C Le Ravallec edits., RAC/SPA publ., Tunis 300p.

UNEP-MAP-RAC/SPA (2006) Proceedings of the second Mediterranean symposium on marine vegetation (Athens, 12-13 December 2003). RAC-SPA publ. Tunis pp. 255.

Urra J, Ramirez AM, Marina P, Salas C, Gofas S, Rueda JL (2013) Highly diverse molluscan assemblages of *Posidonia oceanica* meadows in northwestern Alboran Sea (W Mediterranean): Seasonal dynamics and environmental drivers. Estuarine, Coastal and Shelf Science 117: 136-147.

Žuljević A, Barić Sandro A, Lemac E, Dragutin T, Zekanović H, et al. (2011) Diving in the most amazing part of the Mediterranean: Diving guide for the Šibenik – Knin County. In: Babačić Ajduk A, Škunca O (edts). Public Institution for Management of Nature Protected Areas in Šibenik Knin County pp. 128.

Websites

Project LIFE Posidonia website, Balearic Islands, Spain

http: lifeposidonia.caib.es/user/index_en.htm (Last accessed December 2012)

Project LIFE Posidonia website, Andalucia, Spain

[http://www.lifeposidoniandalucia.es](http://www.lifeposidoniandalucia.es/) (Last accessed December 2012)

Ministro dell’ ambiente (Italy) [ftp://ftp.dpn.minambiente.it/Natura2000/Secondo%20Rapporto%20Dir%20Habitat/Habitat/Habitat_1120.htm](ftp://ftp.dpn.minambiente.it/Natura2000/Secondo Rapporto Dir Habitat/Habitat/Habitat_1120.htm) (Last accessed May 2013)

UNEP WCMC

<http://datadownload.unep-wcmc.org/download/>(Last accessed June 2012)

Join Nature Conservation Committee (JNCC) EUSeaMap project-Mapping European seabed habitats <http://jncc.defra.gov.uk/page-5020> (Last accessed June 2012)

Other sources

*Compilation of data from national projects/reports*

Croatia: Andrej Jaklin – author

Cyprus: Marina Argyrou – Department of Fisheries and Marine Research (DFMR), Cyprus

Egypt: Soha Shabaka – National Institute of Oceanography and Fisheries, Egypt

Greece: Panos Panayotidis, Sylvaine Giakoumi, Vasilis Gerovasileiou, Maria Sini – authors

Libya: Ameer Abdulla – author

Malta: Patrick Schembri – Department of Biology, University of Malta, Malta

Turkey: Melih Ertan Çinar, Ergun Taskin – authors

*Diving clubs and divers*

Aquatours Almeria, Spain: [www.aquatoursalmeria.es](https://hermes.aegean.gr/owa/redir.aspx?C=886b30f2e45142068be4fa1414a7e9be&URL=http%3A%2F%2Fwww.aquatoursalmeria.es)

Barbas Dimitris – Corfu Dive Club, Greece: www.corfudiveclub.com

Baxevanidis Stavros – Go extreme Dive Club, Greece: [www.goextreme.gr](http://www.goextreme.gr/)

Gund Jeanine – Rederis Plongée, France: [www.rederis.com](http://www.rederis.com/)

Kurt Leidl – Najada Diving, Croatia: www.najada.com

Miljenko Marukić – Diving Club MM-Sub, Croatia: [www.mm-sub.hr](http://www.mm-sub.hr/)

Antoniadis Germanos – Diver, Greece

Giourgis Ektoras – Diver, Greece

Pantazis Alekos – Diver, Greece

Poursanidis Dimitris – Marine Ecologist/Diver, Greece

**Sources of data on the distribution of coralligenous formations**

Scientific and grey literature

Abbiati M, Airoldi L, Costantini F, Fava F, Ponti M, et al. (2009) Spatial and temporal variation of assemblages in Mediterranean coralligenous reefs. In: UNEP-MAP-RAC/SPA, 2009. Proceedings of the 1^st^ symposium on conservation of the coralligenous bio-concretions (Tabarka 16–19 January). Pergent-Martini C, Brichet M (eds) RAC/SPA publ. Tunis pp. 269.

Aktan Y (2012) On the occurrence of coralligenous algae in the Johnston Bank (Aegean Sea). Journal of the Black Sea / Mediterranean Environment 18(3): 414–419.

Angiolillo M, Canese S, Salvati E, Giusti M, Cardinali A, et al. (2009) Presence of *Corallium rubrum* on coralligenous assemblages below 50 m along Calabrian coast (South Italy). In: UNEP-MAP-RAC/SPA, 2009. Proceedings of the 1^st^ symposium on conservation of the coralligenous bio-concretions (Tabarka 16–19 January). Pergent-Martini C, Brichet M (eds) RAC/SPA publ. Tunis pp. 269.

Angiolillo M, Canese S, Giusti M, Bo M, Cardinali A, et al. (2009) Distribution of *Acanthogorgia cf. hirsuta* along the Calabrian coast (Southern Tyrrhenian Sea, Italy). In: UNEP-MAP-RAC/SPA, 2009. Proceedings of the 1^st^ symposium on conservation of the coralligenous bio-concretions (Tabarka 16–19 January). Pergent-Martini C, Brichet M (eds) RAC/SPA publ. Tunis pp. 269.

Angiolillo M, Bo M, Bavestrello G, Giusti M, Salvati E, et al. (2012) Record of *Ellisella paraplexauroides* (Anthozoa: Alcyonacea: Ellisellidae) in Italian waters (Mediterranean Sea). Marine Biodiversity Records, 5, e4 doi:10.1017/S1755267211000972.

Antoniadou C (2003) Structure of hard substrate benthic assemblages at the lower infralittoral zone in the north Aegean Sea. Doctorate Thesis, Thessaloniki, Greece, pp. 446.

Arko-Pijevac M, Kirinčić, Kovačić M, Benac C (2007) Ssak island (north Adriatic sea): Possible protected marine area. Rapport du Commission International de la Mer Méditerranee 38: 654.

Balata D, Piazzi, Cecchi E, Cinelli F (2005) Variability of Mediterranean coralligenous assemblages subject to local variation in sediment deposition. Marine Environmental Research 60 : 403–421.

Baldacconi R, Corriero G (2009) Effects of the spread of the alga Caulerpa racemosa var. cylindracea on the sponge assemblage from coralligenous concretions of the Apulian coast (Ionian Sea, Italy). Marine Ecology 30: 337–345.

Bakran-Petricioli T, Petricioli D, Radovic J (2009) Challenges and perspectives of calcareous bioconcretions inventory in Croatia. In: UNEP-MAP-RAC/SPA, 2009. Proceedings of the 1st symposium on conservation of the coralligenous bio-concretions (Tabarka 16–19 January). Pergent-Martini C, Brichet M (eds) RAC/SPA publ. Tunis pp. 269.

Bally M, Garrabou J (2007) Thermodependent bacterial pathogens and mass mortalities in temperate benthic communities: a new case of emerging disease linked to climate change. Global Change Biology 13: 2078–2088.

Ben Mustapha K, Komatsu T, Hattour A, Sammari Ch, Zarrouk S, et al. (2002) Tunisian mega benthos from infra (*Posidonia* meadows) and circalittoral (Coralligenous) sites. Bulletin de L’Institut National des Sciences et Techonlogies de Salammbô 29: 23–36.

Ben Mustapha K, Boury-Esnault N, Kartas F, El Abed A, Zarrouk S, et al. (2003) Sponge diversity in the Tunisian waters. Società Italiana di Biologia Marina, 34th Congress.

Ben Mustapha K, Afli A (2007) Quelques traits de la biodiversité marine de Tunisie: Proposition d’aires de conservation et de gestion. Report of the MedSudMed Expert Consultation on Marine Protected Areas and Fisheries Management. MedSudMed Technical Documents. Rome (Italy). pp. 32–55.

Belbacha S, Semroud R, Dupuy de la Grandrive R, Foulquie M (2009) Donnees preliminaires sur la repartition et la composition de la biocenose du coralligene du littoral d’El Kala (Algerie). In: UNEP-MAP-RAC/SPA, 2009. Proceedings of the 1^st^ symposium on conservation of the coralligenous bio-concretions (Tabarka 16–19 January). Pergent-Martini C, Brichet M (eds) RAC/SPA publ. Tunis pp. 269.

Belbacha S, Ramos Espla A, Semroud R (2012) Distribution, composition and state of the coralligensou from Taza (SW Mediterranean, East Algeria). Creation of the first Marine Protected Area. The 2012 Forum of Marine Protected Areas in the Mediterranean, Antalya-Turkey, 25-28 November 2012.

Belsher T, Houlgatte E, Boudouresque CF (2003) Cartographie de la prairie a *Posidonia oceanica* et des principaux facies sedimentaires marins du Parc National de Port-Cros (Var, France, Mediterranee). UNEP-MAP-RAC/SPA, 2006. Proceedings of the second Mediterranean symposium on marine vegetation (Athens, 12-13 December 2003). RAC-SPA publ. Tunis pp. 255.

Benhissoune S, Rais C, Tunesi L, Bazairi H, Haddi S, et al. (2009) Communautes benthiques remarquables du coralligene du Parc National d’Al Hoceima (Mediterranee - Maroc). In: UNEP-MAP-RAC/SPA, 2009. Proceedings of the 1^st^ symposium on conservation of the coralligenous bio-concretions (Tabarka 16–19 January). Pergent-Martini C, Brichet M (eds) RAC/SPA publ. Tunis pp. 269.

Bianchi CN, Morri C, Navone A (2010) I popolamenti delle scogliere rocciose sommerse dell’Area Marina Protetta di Tavolara Punta Coda Cavallo (Sardegna nord-orientale). Travaux scientifiques du Parc national de Port-Cros 24: 39–85.

Bianchi CN, Parravicini V, Montefalcone M, Rovere A, Morri C (2012) The challenge of managing marine biodiversity: A practical toolkit for a cartographic, territorial approach. Diversity 4: 419–452.

Bittar B, Zibrowius H (1997) Scleractinian corals from Lebanon, Eastern Mediterranean, including a non-lesspsian invading species (Cnidaria: Scleractinia). Scientia Marina 61(2): 227–231.

Blouet S, Foulquie M, Dupuy de la Grandrive R (2006) Restauration naturelle des populations de gorgones blanches *Eunicella singularis* (Esper, 1794) après installation d’ancrages écologiques Harmony, sur le site de plongée des Tables. Site Natura 2000 «Posidonies du Cap d’Agde». Défi territorial marin ELGA. ADENA - Association de Défense de l’Environnement et de la Nature des pays d’Agde pp. 50 (Available at: [http://www.adena-bagnas.com](http://www.adena-bagnas.com/) last accessed January 2013).

Blouet S, Dupuy de la Grandrive R, Foulquie M, Lenfant P (2009) Les formations de type «coralligene de plateau» dans les eaux agathoises (Herault, France). Site Natura 2000 FR 910 1414 « Posidonies du Cap d’Agde ». Donnees preliminaires. In: UNEP-MAP-RAC/SPA, 2009. Proceedings of the 1^st^ symposium on conservation of the coralligenous bio-concretions (Tabarka 16–19 January). Pergent-Martini C, Brichet M (eds) RAC/SPA publ. Tunis pp. 269.

Bo M, Bavestrello G, Canese S, Giusti M, Angiolillo M, et al. (2011) Coral assemblage off the Calabrian Coast (South Italy) with new observations on living colonies of *Antipathes dichotoma*. Italian Journal of Zoology 78(2): 231–242.

Bo M, Bavestrello G, Canese S, Giusti M, Salvati E, et al. (2009) Characteristics of a black coral meadow in the twilight zone of the central Mediterranean Sea. Marine Ecology Progress Series 397: 53–61.

Bo M, Bertolino M, Borghini M, Castellano M, Covazzi Harriague A, et al. (2011) Characteristics of the mesophotic megabenthic assemblages of the Vercelli Seamount (North Tyrrhenian Sea). PLoS ONE 6(2): e16357. doi:10.1371/journal.pone.0016357.

Bo M, Di Camillo CG, Puce S, Canese S, Giusti M, et al. (2011) A tubulariid hydroid associated with anthozoan corals in the Mediterranean Sea. Italian Journal of Zoology 78(4): 487–496.

Bo M, Canese S, Spaggiari C, Pusceddu A, Bertolino M, et al. (2012) Deep coral oases in the South Tyrrhenian Sea. PLoS ONE 7(11): e49870. doi:10.1371/journal.pone.0049870

Bramanti L, Vielmini I, Rossi S, Stolfa S, Santangelo G (2011) Involvement of recreational scuba divers in emblematic species monitoring: The case of Mediterranean red coral (*Corallium rubrum*). Journal for Nature Conservation 19: 312–318.

Bruckner AW, Roberts GG (eds) (2009) Proceedings of the First International Workshop on Corallium Science, Management, and Trade. NOAA Technical Memorandum NMFS-OPR-43 and CRCP-8, Silver Spring, MD pp. 153.

Bussoletti E, Cottingham D, Bruckner A, Roberts G, Sandulli R (eds) (2010) Proceedings of the International Workshop on Red Coral Science, Management, and Trade: Lessons from the Mediterranean. NOAA Technical Memorandum CRCP-13, Silver Spring, MD pp. 233.

Campolmi M, Caddeo R, Soro MC, Doneddu (2010) Red coral (*Corallium rubrum* L., 1758) management strategies in Sardinian coastal waters. In: Bussoletti E, Cottingham D, Bruckner A, Roberts G, Sandulli R (eds). Proceedings of the International Workshop on Red Coral Science, Management, and Trade: Lessons from the Mediterranean. NOAA Technical Memorandum CRCP-13, Silver Spring, MD pp. 233.

Cannas R, Caocci F, Follesa MC, Grazioli E, Pedoni C, et al (2010) Multidisciplinary data on the status of red coral (*Corallium rubrum*) resource in Sardinian seas (Central Western Mediterranean). In: Bussoletti E, Cottingham D, Bruckner A, Roberts G, Sandulli R (eds). Proceedings of the International Workshop on Red Coral Science, Management, and Trade: Lessons from the Mediterranean. NOAA Technical Memorandum CRCP-13, Silver Spring, MD pp. 233.

Calderón I, Garrabou J, Aurelle D (2006) Evaluation of the utility of COI and ITS markers as tools for population genetic studies of temperate gorgonians. Journal of Experimental Marine Biology and Ecology 336: 184–197.

Cardinali A, Canese S, Giusti M, Angiolillo M, Salvati E, et al. (2009) Observation of fish assemblages in coralligenous habitats along the Calabrian coast by means of a remotely operated vehicle. In: UNEP-MAP-RAC/SPA, 2009. Proceedings of the 1^st^ symposium on conservation of the coralligenous bio-concretions (Tabarka 16–19 January). Pergent-Martini C, Brichet M (eds) RAC/SPA publ. Tunis pp. 269.

Casellato S, Masiero L, Sichirollo E, Soresi S (2007) Hidden secrets of the Northern Adriatic: “Tegnúe”, peculiar reefs. Central European Journal of Biology 2(1): 122–136.

Casellato S, Stefanon A (2008) Coralligenous habitat in the northern Adriatic Sea: an overview. Marine Ecology 29: 321–341.

Castritsi-Catharios J, Miliiou H, Kapiris K, Kefalas E (2011) Recovery of the commercial sponges in the central and southeastern Aegean Sea (NE Mediterranean) after an outbreak of sponge disease. Mediterranean Marine Science 12(1): 5–20.

Cebrian E, Linares C, Marschal C, Garrabou J (2012) Exploring the effects of invasive algae on the persistence of gorgonian populations. Biological Invasions 14: 2647–2656.

Cerrano C, Arillo A, Azzini F, Calcinai B, Castellano L, et al. (2005) Gorgonian population recovery after a mass mortality event. Aquatic Conservation: Marine and Freshwater Ecosystems 15: 147–157.

Cerrano C, Danovaro R, Gambi C, Pusceddu A, Riva A, et al. (2010) Gold coral (*Savalia savaglia*) and gorgonian forestsenhance benthic biodiversity and ecosystem functioning in the mesophotic zone. Biodiversity Conservation 19: 153–167.

Coma R, Linares C, Ribes M, Diaz D, Garrabou J, et al. (2006) Consequences of a mass mortality in populations of *Eunicella singularis* (Cnidaria: Octocorallia) in Menorca (NW Mediterranean). Marine Ecology Progress Series 327: 51–60.

Coppo S, Diviacco G, Tunesi L (2009) Environmental and conservation relevance of the Punta Manara coralligenous beds (Eastern Ligurian Sea). In: UNEP-MAP-RAC/SPA, 2009. Proceedings of the 1^st^ symposium on conservation of the coralligenous bio-concretions (Tabarka 16–19 January). Pergent-Martini C, Brichet M (eds) RAC/SPA publ. Tunis pp. 269.

Corriero G, Gherardi M, Giangrande A, Longo C, Mercurio M, et al. (2004) Inventory and distribution of hard bottom fauna from the marine protected area of Porto Cesareo (Ionian Sea): Porifera and Polychaeta. Italian Journal of Zoology 71(3): 237–245.

Costantini F, Fauvelot C, Abbiati M (2007) Fine-scale genetic structuring in *Corallium rubrum*: evidence of inbreeding and limited effective larval dispersal. Marine Ecology Progress Series 340: 109–119.

Costantini F, Taviani M, Remia A, Pintus E, Schembri PJ, et al. (2009) Deep-water *Corallium rubrum* (L., 1758) from the Mediterranean Sea: preliminary genetic characterization. Marine Ecology 31(2): 261–269.

Costantini F, Rossi S, Pintus E, Cerrano C Gili JM, et al. (2011) Low connectivity and declining genetic variability along a depth gradient in *Corallium rubrum* populations. Coral Reefs 30: 991–1003.

Cupido R, Cocito S, Sgorbini S, Bordone A, Santangelo G (2008) Response of a gorgonian (*Paramuricea clavata*) population to mortality events: recovery or loss? Aquatic Conservation: Marine and Freshwater Ecosystems 18: 984–992.

Derbal F, Kara H (2009) Les gorgonacea et les alcyonanea des cotes de l’est Algerrien: diversite et etat d’exploitation de *Corallium rubrum.* In: UNEP-MAP-RAC/SPA, Proceedings of the 1^st^ symposium on conservation of the coralligenous bio-concretions (Tabarka 16–19 January). Pergent-Martini C, Brichet M (eds) RAC/SPA publ. Tunis pp. 269.

Deter J, Descamp P, Ballesta L, Boissery P, Holon F (2012) A preliminary study toward an index based on coralligenous assemblages for the ecological status assessment of Mediterranean French coastal waters. Ecological Indicators 20: 345–352.

Falace A, Kaleb S (2011) First contribution to the knowledge of coralline algae distribution in the Slovenian circalittoral zone (Northern Adriatic). Annales Series Historia Naturalis 21(1): 27–40.

Fava F, Ponti M, Abbiati M (2009) Coralligenous assemblages in the northern Adriatic continental shelf. In: UNEP-MAP-RAC/SPA. Proceedings of the 1^st^ symposium on conservation of the coralligenous bio-concretions (Tabarka 16–19 January). Pergent-Martini C, Brichet M (eds) RAC/SPA publ. Tunis pp. 269.

Fava F, Bavestrello G, Valisano L, Cerrano C (2010) Survival, growth and regeneration in explants of four temperate gorgonian species in the Mediterranean Sea. Italian Journal of Zoology 77(1): 44–52.

Ferdeghini F, Acunto S, Cocito S, Cinelli F (2000) Variability at different spatial scales of a coralligenous assemblage at Giannutri Island (Tuscan Archipelago, northwest Mediterranean). Hydrobiologia 440: 27–36.

Ferrier-Pagès, Tambutté E, Zamoum T, Segonds N, Merle P-L, et al. (2009) Physiological response of the symbiotic gorgonian *Eunicella singularis* to a long-term temperature increase. The Journal of Experimental Biology 212: 3007–3015.

Freiwald A, Beuck L, Rüggeberg A, Taviani M, Hebbeln D et al. (2009) The white coral community in the Central Mediterranean Sea revealed by ROV surveys. Oceanography 22(1): 58–74.

Gatti G, Montefalcone M, Rovere A, Parravicini V, Morri C, et al. (2012) Seafloor integrity down the harbor waterfront: the coralligenous shoals off Vado Ligure (NW Mediterranean). Advances in Oceanography and Limnology 3(1): 51–67.

Georgiadis M, Papatheodorou G, Tzanatos E, Geraga M, Ramfos A, et al. (2009) Coralligène formations in the eastern Mediterranean Sea: Morphology, distribution, mapping and relation to fisheries in the southern Aegean Sea (Greece) based on high-resolution acoustics. Journal of Experimental Marine Biology and Ecology 368: 44–58.

Giangrande A, Delos AL, Fraschetti S, Musco L, Licciano M, et al (2003) Polychaete assemblages along a rocky shore on the South Adriatic coast (Mediterranean Sea): patterns of spatial distribution. Marine Biology 143: 1109–1116

Giaccone G (2007) Il Coralligeno come paesaggio marino sommerso: Distribuzione sulle coste Italiane. Biologia Marina Mediterranea 14(2): 126–143.

Gili JM, Ros J (1984) Study and cartography of the benthic communities of Medes Islands (NE Spain). P.S.Z.N.I: Marine Ecology 6(3): 219–238.

Girosi L, Ramoino P, Diaspro A, Gallus L, Ciarcia G, et al. (2005) FMRFamide-like immunoreactivity in the sea-fan *Eunicella cavolini* (Cnidaria: Octocorallia). Cell Tissue Research 320: 331–336.

Giuliani S, Virno Lamberti C, Sonni C, Pellegrini D (2005) Mucilage impact on gorgonians in the Tyrrhenian Sea. Science of the Total Environment 353: 340–349.

Giusti M, Canese S, Angiolillo M, Bo M, Salvati E, et al. (2009) Three-dimensional distribution of *Gerardia savaglia* in relation to depth, orientation and slope of the substrata in the south Tyrrhenian Sea. In: UNEP-MAP-RAC/SPA. Proceedings of the 1^st^ symposium on conservation of the coralligenous bio-concretions (Tabarka 16–19 January). Pergent-Martini C, Brichet M (eds) RAC/SPA publ. Tunis pp. 269.

Gori A (2011) The ecology of deep sublittoral populations of Mediterranean gorgonians. PhD Thesis. Universitat de Barcelona pp. 170.

Greenpeace (2012) I tesori sommersi del canale di Sicilia pp. 26 (Available at: [www.greenpeace.org](http://www.greenpeace.org/). Last accessed March 2013).

Harmelin J-G, Garrabou J (2005) Suivi d’une population de *Paramuricea clavata* (Risso, 1826) (Cnidaria, Octocorallia, Gorgonacea) dans le Parc National de Port-Cros (Méditerranée, France) : Comparaison des états 1992 et 2004 sur le site de la Galère. Scientific Reports Port-Cros National Park 21: 175–191.

Harmelin J-G, Bitar G, Zibrowius H (2009) Smittinidae (Bryozoa, Cheilostomata) from coastal habitats of Lebanon (Mediterranean Sea), including new and non-indigenous species. Zoosystema 31 (1): 163–187.

Ioannou E, Abdel-Razika AF, Zervou M, Christofidis D, Alexid X, et al. (2009) 5α,8α-Epidioxysterols from the gorgonian *Eunicella cavolini* and the ascidian *Trididemnum inarmatum*: Isolation and evaluation of their antiproliferative activity. Steroids 74: 73–80.

Issaris Y, Katsanevakis S, Pantazi M, Vassilopoulou V, Panayotidis P, et al. (2012) Ecological mapping and data quality assessment for the needs of ecosystem-based marine spatial management: case study Greek Ionian Sea and the adjacent gulfs. Mediterranean Marine Science 13(2): 297–311.

IUCN (2011). Towards a representative network of Marine Protected Areas in Libya. Gland, Switzerland and Málaga, Spain: IUCN pp. 68.

Kipson S, Novosel M, Radić I, Kružić P, Požar-Domac A (2009) The biodiversity of macrobenthos within the coralligenous community dominated by the red gorgonian Paramuricea clavata in the central part of the Eastern Adriatic Sea (Croatia): preliminary results. In: UNEP-MAP-RAC/SPA, 2009. Proceedings of the 1^st^ symposium on conservation of the coralligenous bio-concretions (Tabarka 16–19 January). Pergent-Martini C, Brichet M (eds) RAC/SPA publ. Tunis pp. 269.

Kipson S, Fourt M, Teixidó N, Cebrian E, Casas E, et al. (2011) Rapid biodiversity assessment and monitoring method for highly diverse benthic communities: A case study of Mediterranean coralligenous outcrops. PLoS ONE 6(11):e27103. doi:10.1371/journal.pone.0027103

Kontiza I, Abatis D, Malkate K, Vagias C, Roussis V (2006) 3-Keto steroids from the marine organisms *Dendrophyllia cornigera* and *Cymodocea nodosa*. Steroids 71(2): 177–181.

Kružić P (2002) Marine fauna of the Mljet National Park (Adriatic Sea, Croatia). 1. Anthozoa. Natura Croatica 11(3): 265–292.

Kružić P (2007) Anthozoan fauna of Telašćica Nature Park (Adriatic Sea, Croatia). Natura Croatica. 16(4): 233–266.

Kružić P (2008) First records of *Cladopsammia rolandi* (Cnidaria: Anthozoa) in the Adriatic Sea. Natura Croatica 17(1): 9–14.

Kruzic P, Zibrowius H, Pozar-Domac A (2002) Actiniaria and Scleractinia (Cnidaria, Anthozoa) from the Adriatic Sea (Croatia): First records, confirmed occurrences and significant range extensions of certain species. Italian Journal of Zoology 69(4): 345–353.

Ledoux J-B (2010) Biologie de la conservation du corail rouge, *Corallium rubrum* (Linnaeus, 1758): Impact du changement global sur l’évolution des populations infralittorales en Méditerranée Nord-Occidentale. Thèse de Doctorat de l’Université de la Méditerranée pp. 281.

Linares C, Coma R, Diaz D, Zabala M, Hereu B, et al. (2005) Immediate and delayed effects of a mass mortality event on gorgonian population dynamics and benthic community structure in the NW Mediterranean Sea. Marine Ecology Progress Series 305: 127–137.

Linares C (2006) Population ecology and conservation of a marine long-lived species: the red gorgonian *Paramuricea clavata*. PhD Thesis. Universitat de Barcelona pp. 232.

López E, San Martín G, Jimenez M (1996) Syllinae (Syllidae, Annelida, Polychaeta) from Chafarinas lslands (Alborán Sea, W Mediterranean). Miscèllania Zoològica 19(1): 105–118.

López-González PJ, Megina C, Martínez I, Gómez G, Arroyo MC, et al. (2010) The northern distributional limits of *Dendrophyllia laboreli* (Cnidaria: Scleractinia: Dendrophylliidae). Marine Biodiversity Records 3: 1–4.

Maldonado M (1992). Demosponges of the red coral bottoms from the Alboran Sea. Journal of Natural History 26: 1131–1161.

Maldonado M, López-Acosta M, Sánchez-Tocino L, Sitjà C (2013) The rare, giant gorgonian *Ellisella paraplexauroides*: demographics and conservation concerns. Marine Ecology Progress Series 479: 127–141.

Mastronuzzi G, Pignatelli C, Sansò P, Selleri G, Caputo R, et al. (2008) Geological, geodynamic and morphological features of Apulia (Italy). In: Mastronuzzi G, Sansò P, Brückner H, Vött A, Pignatelli C, et al. (eds). Palaeotsunami imprints along the coasts of the Central Mediterranean Sea. Field Guide. Ostuni (Italy) and Ionian Islands (Greece): 2nd International Tsunami Field Symposium. IGCP Project 495. GI2S Coast Research Pubblication. pp. 15–36.

Mastrototaro F, Onghia GD, Corriero G, Matarrese A, Maiorano P, et al (2010) Biodiversity of the white coral bank off Cape Santa Maria di Leuca (Mediterranean Sea): An update. Deep-Sea Research 57: 412–430.

Mokhtar-Jamaï K, Pascual M, Ledoux J-B, Coma R, Féral P, et al. (2011) From global to local genetic structuring in the red gorgonian *Paramuricea clavata*: the interplay between oceanographic conditions and limited larval dispersal. Molecular Ecology 20(16): 3291–3305.

Mistri M, Ceccherelli VU (1994) Growth and secondary production of the Mediterranean gorgonian *Paramuricea clavata* (Risso, 1826). Marine Ecology Progress Series 103: 291–296.

Mistri M (1995) Population structure and secondary production of the Mediterranean octocoral *Lophogorgia ceratophyta* (L. 1758). P.S.Z.N.I: Marine Ecology 16(3): 181–188.

Morri C, Vafidis D, Peirano A, Chintiroglou CC, Bianchi CN (2000) Anthozoa from a subtidal hydrothermal area of Milos Island (Aegean Sea), with notes on the construction potential of the scleractinian coral *Madracis pharensis.* Italian Journal of Zoology 67: 319–325.

Novosel M, Bakran-Petricioli B, Požar-Domac A, Kružić P, Radić I (2002) The benthos of the northern part of the Velebit Channel (Adriatic Sea, Croatia). Natura Croatica 11(4): 387–409.

Novosel M, Požar-Domac A, Pasarić M (2004) Diversity and distribution of the bryozoa along underwater cliffs in the Adriatic Sea with special reference to thermal regime. P.S.Z.N. Marine Ecology 25(2): 155–170.

Ocãna A, Sánchez Tocino L, López-González PJ (2000) Faunistic and biogeographical observations concerning the Anthozoa (Cnidaria: Anthozoa) of the Granada coast (Sea of Alboran). Zoologica Baetica 11: 51–65.

OCEANA (2008) Propuesta de áreas marinas de importancia ecológica: Atlántico sur y Mediterráneo Español. OEANA y Fundación Biodiversidad pp. 132.

OCEANA (2008) Estudio bionómico de Cabrera: Estudio bionómico de los fondos profundos del Parque Nacional Maritimo Terrestre del Archipiélago de Cabrera y sus Alrededores. OCEANA y Govern de les Illes Balears pp. 60.

Pedoni C, Follesa MC, Cannas R, Matta G, Pesci P, et al (2009) Preliminary data on red coral *Corallium rubrum* (Linneo, 1758) population of Sardinian Sea (Western Mediterranean). In: UNEP-MAP-RAC/SPA. Proceedings of the 1^st^ symposium on conservation of the coralligenous bio-concretions (Tabarka 16–19 January). Pergent-Martini C, Brichet M (eds) RAC/SPA publ. Tunis pp. 269.

Peña Cantero AL, García Carrascosa AM (2002) The benthic hydroid fauna of the Chafarinas Islands (Alborán Sea, western Mediterranean). Zoologische Verhandelingen (Zoologische Verhandelingen Leiden) 337: 1–180.

Piazzi L, Pardi G, Cinelli F (1999) Algal vertical zonation and seasonal dynamics along a subtidal cliff on Gorgona Island (Tuscan archipelago, Italy). Plant Biosystems - An International Journal Dealing with all Aspects of Plant Biology: Official Journal of the Societa Botanica Italiana 133(1): 3–13.

Piazzi L, Balata D, Cecchi E, Cinelli F, Sartoni G. (2009) Species composition and patterns of diversity of macroalgal coralligenous assemblages in the north-western Mediterranean Sea. Journal of Natural History 44(1): 1–22.

Ponti M, Fava F, Abbiati M (2011) Spatial-temporal variability of epibenthic assemblages on subtidal biogenic reefs in the northern Adriatic Sea. Marine Biology 158(7): 1447–1459.

Ribes M, Coma R, Rossi S (2003) Natural feeding of the temperate asymbiotic octocoral-gorgonian *Leptogorgia sarmentosa* (Cnidaria: Octocorallia). Marine Ecology Progress Series 254: 141–150.

Rossi S, Tsounis G (2007) Temporal and spatial variation in protein, carbohydrate, and lipid levels in Corallium rubrum (Anthozoa, Octocorallia). Marine Biology 152: 429–439.

Rossi S, Tsounis G, Orejas C, Padrón T, Gili JM, et al. (2008) Survey of deep-dwelling red coral (*Corallium rubrum*) populations at Cap de Creus (NW Mediterranean). Marine Biology 154: 533–545.

Rosso A, Sanfilippo R (2009) The contribution of bryozoans and serpuloideans to coralligenous concretions from SE Sicily. In: UNEP-MAP-RAC/SPA. Proceedings of the 1^st^ symposium on conservation of the coralligenous bio-concretions (Tabarka 16–19 January). Pergent-Martini C, Brichet M (eds) RAC/SPA publ. Tunis pp. 269.

Rouanet E, Lelong P, Lecalard C, Martin Y (2009) Estimation of coralligenous assemblages ecological state around Embiez archipelago (Var, France). In: UNEP-MAP-RAC/SPA. Proceedings of the 1^st^ symposium on conservation of the coralligenous bio-concretions (Tabarka 16–19 January). Pergent-Martini C, Brichet M (eds) RAC/SPA publ. Tunis pp. 269.

Rovere A, Ferraris F, Parravicini V, Navone A, Morri C, et al. (2013) Characterization and evaluation of a marine protected area: ‘Tavolara – Punta Coda Cavallo’ (Sardinia, NW Mediterranean). Journal of Maps, doi:10.1080/17445647.2013.778081.

Ruci S, Gogo S, Beqiraj S, Kashta L (2012) Natural values of the Sazani island – Karaburuni peninsula, as good tools and indicators for the conservation and management of the first MPA of Albania. The 2012 Forum of Marine Protected Areas in the Mediterranean, Antalya – Turkey, 25–28 November.

Russo A (1985) Ecological observations on the gorgonian sea fan *Eunicella cavolinii* in the Bay of Naples. Marine Ecology Progress Series 24: 155–159.

Salomidi M, Smith C, Katsanevakis S, Panayotidis P and Papathanassiou V (2009) Some observations on the structure and distribution of gorgonian assemblages in the eastern Mediterranean Sea. In: UNEP-MAP-RAC/SPA. Proceedings of the 1^st^ symposium on conservation of the coralligenous bio-concretions (Tabarka 16–19 January). Pergent-Martini C, Brichet M (eds) RAC/SPA publ. Tunis pp. 269.

Salomidi M, Zibrowius H, Issaris Y, Milionis K (2010) *Dendrophyllia* in Greek waters, Mediterranean Sea, with the first record of *D. ramea* (Cnidaria, Scleractinia) from the area. Mediterranean Marine Science 11(1): 189–194.

Santangelo G, Carletti E, Maggi E, Bramanti L (2003) Reproduction and population sexual structure of the overexploited Mediterranean red coral *Corallium rubrum*. Marine Ecology Progress Series 248: 99–108.

Santangelo G, Cupido R, Cocito S, Bramanti L, Tsounis G, et al. (2012) Proceedings of the 12th International Coral Reef Symposium, Cairns, Australia, 9-13 July.

Sarà M (1968) Un coralligeno di piattaforma (coralligène de plateau) lungo il littorale Pugliese. Archivio di Oceanografia e Limnologia 15 (suppl.): 139–150.

Sarà M (1969) Research on coralligenous formations : problems and perspectives. Pubblicazioni della Stazione Zoologica di Napoli 37(suppl.): 124–134.

Sarà M (1971) Le peuplement du coralligène des Pouilles. Rapport du Commission International de la Mer Méditerranee 20(3): 235–237.

Sarà, (1967) Researches on bottom organisms, ecology and environmental biology of Adriatic Coast. Institute of Zoology and Comparative Anatomy, Final report, pp46.

Sartoni G, Rossi S (1998) New records for the benthic algal flora of the Northern Adriatic Sea. Flora Mediterranea 8: 9–16.

Sartoretto S, Francour P (2012) Bathymetric distribution and growth rates of *Eunicella verrucosa* (Cnidaria: Gorgoniidae) populations along the Marseilles coast (France). Scientia Marina 76(2): 349–355.

**Skoufas G** (2006) Comparative biometry of Eunicella singularis (Gorgonian) sclerites at East Mediterranean Sea (North Aegean Sea, Greece). Marine Biology 149(6): 1365–1370.

Smith C, Sakellariou D, McCoy F, Wachsmann S (2009) Deep coral environments south of Crete. Proceedings of the 9th Symposium on Oceanography & Fisheries, Patra, Greece 1: 665–668.

Templado J, García-Carracosa M, Baratech L, Capaccioni R, Juan A, et al. (1986) Estudio preliminar de la fauna asociada a los fondos coralíferos del mar de Alborán (SE de España). Boletíno del Instituto Español de Oceanografía 3(4): 93–104.

Terlizzi A, Scuderi D, Fraschetti S, Guidetti P, Boero F (2003) Molluscs on subtidal cliffs: patterns of spatial distribution. Journal of the Marine Biological Association of the United Kingdom 83: 165–172.

Trygonis V, Sini M (2012) photoQuad: A dedicated seabed image processing software, and a comparative error analysis of four photoquadrat methods. Journal of Experimental Marine Biology and Ecology 424-425: 99–108.

Tsounis G (2005) Demography, Reproductive Biology and Trophic Ecology of Red Coral (*Corallium rubrum* L.) at the Costa Brava (NW Mediterranean): Ecological Data as a Tool for Management. PhD Thesis. University of Bremen pp. 106.

Tsounis G, Rossi S, Gili JM, Arntz W (2006) Population structure of an exploited benthic cnidarian: the case study of red coral (*Corallium rubrum* L.). Marine Biology 149: 1059–1070.

Tsounis G, Martinez L, Bramanti L, Viladrich N, Gili JM, et al. (2012) Anthropogenic effects on reproductive effort and allocation of energy reserves in the Mediterranean octocoral *Paramuricea clavata*. Marine Ecology Progress Series 449: 161–172.

Tursi A, Mastrototaro F, Matarrese A, Maiorano P, D’Onghia G (2004) Biodiversity of the white coral reefs in the Ionina Sea (central Mediterranean). Chemistry and Ecology 20: 107–116.

UNEP-MAP RAC/SPA (2005) Evaluation and follow-up of activities carried out within the framework of the MedMPA Project – Lot n°4 Task I and J. Final report, pp. 123.

UNEP-MAP RAC/SPA (2007) Integrated coastal area management in Cyprus: Biodiversity concerns on the Coastal Area Management Program of Cyprus. By Ramos A, Cebrián D, Demetropoulos A (eds). RAC/SPA, Tunis. pp. 69.

UNEP-MAP RAC/SPA (2009) State of knowledge of the geographical distribution of the coralligenous and other calcareous bio-concretions in the Mediterranean. By Agnesi S, Annunziatellis A, Cassese ML, La Mesa G, Mo G, et al. RAC/SPA, Tunis, pp. 167.

UNEP-MAP RAC/SPA (2010) The Mediterranean Sea Biodiversity: state of the ecosystems, pressures, impacts and future priorities. By Bazairi H, Ben Haj S, Boero F, Cebrian D, De Juan S, et al. (eds). RAC/SPA, Tunis, pp. 100.

Urra J, Rueda JL, Gofas S, Marina P, Salas C (2011) A species-rich molluscan assemblage in a coralligenous bottom of the Alboran Sea (south-western Mediterranean): intra-annual changes and ecological considerations. Journal of the Marine Biological Association of the United Kingdom 92(4): 665–677.

Vafidis D, Koukouras A, Voultsiadou-Koukoura E (1994) Octocoral fauna of the Aegean Sea with a checklist of the Mediterranean species: new information, faunal comparisons. Annale de L’Institut océanographique 70: 217–229.

Vafidis D, Koukouras A, Voultsiadou-Koukoura E (1997) Actinaria, corallimorpharia, and scleractinia (Hexacorallia, Anthozoa) of the Aegean Sea, with a checklist of the eastern Mediterranean and Black Sea species. Israel Journal of Zoology 43: 55–70.

Vafidis D (2009) First record of *Leptogorgia sarmentosa* (Octocorallia: Gorgoniidae) from the eastern Mediterranean Sea. Marine Biodiversity Records, 2, e17 doi:10.1017/S1755267208000195.

Voultsiadou E, Vafidis D (2004) Rare sponge (Porifera: Demospongiae) species from the Mediterranean Sea. Journal of Marine Biological Association U.K. 84: 593–598.

Weinbauer M, Velimirov B (1995) Morphological variations in the Mediterranean sea fan *Eunicella cavolini* (Coelenterata: Gorgonacea) in relation to exposure, colony size and colony region. Bulletin of Marine Science 56(1): 283–295.

Zavodnik D, Pallaoro A, Jaklin A, Kovačić, Arko-Pijevac M (2005) A benthos survey of the Senj Archipelago (North Adriatic Sea, Croatia). Acta Adriatica 46(Suppl.2): 3–68.

Zibrowius H (1979) Campagne de la Calypso en Méditerranée nord-orientale (1955, 1956, 1960, 1964). 7. Scléractiniaires. Annales de l'Institut Océanographique 55: 7–28.

Zibrowius H (1980) Les scléractiniaires de la Méditerranée et de l'Atlantique nord-oriental. Mémoires de l'Institut Océanographique, Monaco, 11: 284p.

Žuljević A, Barić Sandro A, Lemac E, Dragutin T, Zekanović H, et al. (2011) Diving in the most amazing part of the Mediterranean: Diving guide for the Šibenik – Knin County. In: Babačić Ajduk A, Škunca O (edts). Public Institution for Management of Nature Protected Areas in Šibenik Knin County pp. 128.

Websites

Agenzia Regionale per la Protezione dell’ Ambiente, Italy

<http://www.arpa.sicilia.it/news.jsp?ID_NEWS=242&GTemplate=default.jsp> (Last accessed April 2013)

Area Marina Protetta Porto Cesareo, Italy

<http://www.ampportocesareo.it/ambiente-marino> (Last accessed March 2013)

Club Nautico Ceuta

[http://www.ceutabuceo.com](http://www.ceutabuceo.com/) (Last accessed May 2013)

Diving in Croatia

<http://www.ronjenjehrvatska.com/en/diving_sites> (Last accessed January 2013)

Isola Tremiti ­– Italy

[http://tremiti.planetek.it](http://tremiti.planetek.it/) (Last accessed March 2013)

Jakl Z, Brajčić D, Baučić M (2009) - Morska bioraznolikost akvatorija uz značajni krajobraz na sjeverozapadnom djelu Dugog otoka, Stručna studija, Udruga Sunce, Split, Project COAST, UNDP

<http://natura-jadera.com/Dokumenti/Dugi_otok_podmorje_zavrsni_izvjesaj.pdf> (last accessed May 2013)

Jakl Z, Brajčić D et al (2010) Kartiranje morskih svojti i staništa Splitsko-dalmatinske županije, Udruga Sunce, Split.

<http://www.sunce-st.org/razno/SD_zupanija_zavrsni_izvjesaj_2011_sijec> (last accessed September 2012)

Janjanin L (2012) Report on the mapping of marine habitats in the Istria Region, IPA Adriatic CBC project SHAPE (*Shaping an Holistic Approach to Protect the Adriatic Environment: between coast and sea*), Institute for Spatial Planning in Istria County

<http://shape.istra-istria.hr/uploads/media/Rezultati_kartiranja.pdf> (last accessed May 2013)

Project EU IPA 2007 “Identification and setting-up of the marine part of Natura 2000 network in Croatia” (Thanks to all participants of project workshops that contributed with their knowledge on important marine habitats including caves and coralligenous communities) Map viewer:

[http://www.natura2000.hr/KartaLokacija.aspx](https://webmail.aegean.gr/owa/redir.aspx?C=8--vXGXDGEaUYfJ2jB-tuHGFDM-VQNBIVcDs0-EkzC_jkHuenFBHR-eYuawrLkEfFtZ-1xY8CDo.&URL=http%3A%2F%2Fwww.natura2000.hr%2FKartaLokacija.aspx) (Last accessed May 2013)

Punta Campanella Area Marina Protetta, Italy

[http://www.puntacampanella.org/index.asp](https://webmail.aegean.gr/owa/redir.aspx?C=JfN6GYcPY0ObBcUMcJK_L7sLfYxFJdBIkqiqEPacxqWnscPcPVaAfk3XwLPEquK8DLuNO6eUXbs.&URL=http%3A%2F%2Fwww.puntacampanella.org%2Findex.asp) (Last accessed March 2013)

SeaMap–Seascape Evaluation Assessment and Mapping

<http://www.seamap.it/?page_id=876> (Last accessed May 2013)

Wannadive.net – World dive site atlas

[http://www.wannadive.net](http://www.wannadive.net/) (Last accessed February 2013)

Žuljević et al (2009) Morska bioraznolikost Biševa i JI strane otoka Visa - stručna podloga za održivo upravljanje, Project COAST, UNDP

[http://issuu.com/undphr/docs/morska_bioraznolikost#embed](https://webmail.aegean.gr/owa/redir.aspx?C=8--vXGXDGEaUYfJ2jB-tuHGFDM-VQNBIVcDs0-EkzC_jkHuenFBHR-eYuawrLkEfFtZ-1xY8CDo.&URL=http%3A%2F%2Fissuu.com%2Fundphr%2Fdocs%2Fmorska_bioraznolikost%23embed) (Last accessed May 2013)

Other sources

*Compilation of data from national projects/reports*

Croatia: Petra Rodic – author

Greece: Maria Sini, Sylvaine Giakoumi – authors

Turkey: Melih Ertan Çinar, Ergun Taskin – authors

*Diving clubs and divers*

Aquatours Almeria: [www.aquatoursalmeria.es](http://www.aquatoursalmeria.es/)

Barbas Dimitris – Corfu Dive Club: [www.corfudiveclub.com](http://www.corfudiveclub.com/)

Kotsifas John – Tech Diving Team: [www.techdivingteam.gr](http://www.techdivingteam.gr/)

Kurt Leidl – Najada Diving: [www.najada.com](http://www.najada.com/)

Sofos Paris – Diving Pelion: [www.divingpelion.gr](http://www.divingpelion.gr/)

Antoniadis Germanos – Diver, Greece

Giourgis Ektoras– Diver, Greece

Poursanidis Dimitris – Marine Ecologist/Diver, Greece

Tsantilas Christos– Diver, Greece

**Sources of data on the distribution of marine caves**

Scientific and grey literature

Arko-Pijevac M, Benac Č, Kovačić M, Kirinčić M (2001) A submarine cave at the island of Krk (North Adriatic Sea). Natura Croatica 10: 163–184.

Bakran-Perticioli Τ, Radolović M, Petricioli D (2012) How diverse is sponge fauna in the Adriatic Sea? Zootaxa 3172: 20–38.

Bakran-Petricioli T, Vacelet J, Zibrowius H, Petricioli D, Chevaldonné P, et al. (2007) New data on the distribution of the ‘deep-sea’ sponges *Asbestopluma hypogea* and *Oopsacas minuta* in the Mediterranean Sea. Marine Ecology: An evolutionary Perspective 28: 10–23.

Bakran-Petricioli, T., and P. Kružić (2002) Marine caves. In: S.Gottstein Matocec (ed.), An overview of the cave and interstitial biota of Croatia. Natura Croatica 11(suppl. 1): 77–79.

Bayarı CS, Ozyurt NN, Oztan M, Bastanlar Y, Varinlioglu G, et al (2011) Submarine and coastal karstic groundwater discharges along the southwestern Mediterranean coast of Turkey. Hydrogeology Journal 19: 399–414.

Belmonte G, Costantini A, Moscatello S, Denitto F, Shkurtaj B (2006) Le grotte sommerse della penisola del Karaburun (Albania): primi dati. Thalassia Salentina 29: 15–28.

Ben Mustapha K, Zarrouk A, Souissi A, El Abed A (2003) Diversité des demosponges tunisiennes. Bulletin de l'Institut National des Sciences et Technologies de la Mer de Salammbô 30: 55–77.

Bianchi CN, Morri C, Navone A (2010) I popolamenti delle scogliere rocciose sommerse dell’Area Marina Protetta di Tavolara Punta Coda Cavallo (Sardegna nord-orientale). Travaux scientifiques du Parc national de Port-Cros 24: 39–86.

Božić V (2005) Špilja Pijavica nekad i sad, Senjski zbornik 32: 483–496.

Chevaldonné P, Lejeusne C (2003) Regional warming-induced species shift in north-west Mediterranean marine caves. Ecology Letters 6: 371–379.

Cicogna F, Bianchi CN, Ferrari G, Forti P (2003) Le grotte marine: cinquant’anni di ricerca in Italia. Roma: Ministero dell’Ambiente e della Tutela del Territorio. 505 p.

Čižmek H, Radolović M, Petricioli D, Dujmović S, Kružić P, et al (2006) Distribution of benthic communities along the light gradient in Brbinjšćica cove, Dugi otok, In: Besendorfer V, Klobučar G, editors. Zagreb: Proceeding of Abstracts of the 9th Croatian Biological Congress. pp. 267–268.

Čižmek H, Zubak I (2009) Nova biološka istraživanja u potopljenim speleo-objektima u uvali Brbinjšćica (Dugi Otok). 20 000 milja, Zadar.

Corriero G, Gherardi M, Giangrande A, Longo C, Mercurio M, et al. (2004) Inventory and distribution of hard bottom fauna from the Marine Protected area of Porto Cesareo (Ionian Sea): Porifera and Polychaeta. Italian Journal of Zoology 71: 237–245.

CREOCEAN-DREAL (2010) Recensement des grottes submergées ou semi-submergées sur le littoral Corse. Rapport final. 80 p.

Cuculic V, Cukrov N, Kwokal Z, Mlakar M (2011) Distribution of trace metals in anchialine caves of Adriatic Sea, Croatia. Estuarine, Coastal and Shelf Science 95: 253–263.

Cukrov M, Cukrov N, Jalžić B, Cuculić V (2007) Geokemijska istraživanja voda anhihaline jame u uvali Bjejajka, otok Mljet. Subterranea Croatica 8: 16–19.

Deidun A, AIS Environmental Ltd (2006) Marine Scientific Surveys around Filfla for its conservation – draft management plan and monitoring report. Malta: Report compiled for AIS Environmental Ltd. 113 p.

Demirkol E, Özyurt NN, Ceylan H, Alparslan B, Kayhan B, et al (2011) Türkiye kıyılarında kıyı-denizaltı magaraları ve tatlısu bosalımı arastırmaları ve 1998-2010 dönemi envanteri, Türk Speoloji Dergisi 2(2): 7–18.

Džaja K (2003) Geomorfološke značajke Dugog otoka. Geoadria 8(2): 5–44.

Fritz F, Bahun S (1997) The morfogensis of submarine springs in the bay of Kastela, Croatia. Geologia Croatica 50(1): 105–110.

Gerovasileiou V, Voultsiadou E (2012) Marine Caves of the Mediterranean Sea: A Sponge Biodiversity Reservoir within a Biodiversity Hotspot. PLoS ONE 7(7): e39873.

Gottstein S, Ivković M, Ternjej I, Jalžić B, Kerovec M (2007) Environmental features and crustacean community of anchihaline hypogean waters on the Kornati islands, Croatia. Marine Ecology - P S Z N I 28 (suppl. 1): 24–30.

Gottstein S, Jalžić B (2007) Biospeleološka istraživanja vodene faune anihalnih špilja i jama na području NP Kornati, Subterranea Croatica 9: 20–30.

Grubelic I, Antolic B, Span A (1998) Benthic flora and fauna in a submarine cave in the central Adriatic Sea. Rapport du Commission International de la Mer Méditerranee 35: 446–447.

Harmelin JG, Bitar G, Zibrowius H (2009) Smittinidae (Bryozoa, Cheilostomata) from coastal habitats of Lebanon (Mediterranean sea), including new and non-indigenous species. Zoosystema 31(1): 163–187.

Harmelin JG, Boury-Esnault N, Fichez R, Vacelet J, Zibrowius H (2003) Peuplement de la grotte sous-marine de l’île de Bagaud (Parc national de Port-Cros, France, Méditerranée). Travaux scientifiques du Parc national de Port-Cros 19: 117–134.

IUCN (2012) Propuesta de una red representativa de áreas marinas protegidas en el mar de Alborán / Vers un réseau représentatif d’aires marines protégées dans la mer d’Alboran. Gland y Málaga: IUCN. 124 p.

Japundžić D (2010) Lavlja jama (Lion's Pit). Katalog uz izložbu, Muzej Grada Crikvenice, Crikvenica.

Jones DA, Knight-Jones EW, Moyse J, Babbage PC, Stebbing ARD (1968) Some biological problems in the Aegean. Underwater Association Report, Malta 1968: 73–78.

Kovač Konrad P, Jalžić V, Buzjak N (2012) Speleo-diving explorations of the Zečica water spring. In: Prpić M, Kovačević A, Kovačev M, Žalac E, Prpić B, editors. Zagreb: Almanac of Summaries of the Croatian Caver’s Annual Meeting 2012. pp. 14–15.

Kovačić M (1999) *Gammogobius steinitzi* Bath, 1971, a fish new to the Adriatic Sea. Natura Croatica 8(1): 1–7.

Kršinić F (2005): *Badijella jalzici* – a new genus and species of calanoid copepod (Calanoida, Ridgewayiidae) from an anchialine cave on the Croatian Adriatic coast. Marine Biology Research 5: 281–289.

Kršinić F (2008): Description of *Speleophria mestrovi* sp. nov., new copepod (Misophrioida) from an anchialine cave in the Adriatic Sea. Marine Biology Research 4: 304–312.

Kružić P (2002) Marine fauna of the Mljet National Park (Adriatic Sea, Croatia). 1. Anthozoa. Natura Croatica 11(3): 265–292.

Kružić P (2007) Anthozoan fauna of Telašćica Nature Park (Adriatic Sea, Croatia). Natura Croatica 16(4): 233–266.

Kružić P (2008) First records of *Cladopsammia rolandi* (Cnidaria: Anthozoa) in the Adriatic Sea. Natura Croatica 17(1): 9–14.

Laborel J, Vacelet J (1959) Les grottes sous-marines obscures en Méditerranée. Comptes Rendus de l'Académie des Sciences 248: 2619–2621.

Ledoux J-B (2010) Biologie de la conservation du corail rouge, *Corallium rubrum* (Linnaeus, 1758): Impact du changement global sur l’évolution des populations infralittorales en Méditerranée Nord-Occidentale. Thèse de Doctorat de l’Université de la Méditerranée 281 p.

Lejeusne C, Chevaldonné P (2006) Brooding crustaceans in a highly fragmented habitat: the genetic structure of Mediterranean marine cave-dwelling mysid populations. Molecular Ecology 15: 4123–4140.

Logan A (2003) Marine fauna of the Mljet National Park (Adriatic Sea, Croatia). 3. Brachiopoda. Natura Croatica 12(4): 233–243.

Logan A, Zibrowius H (1994) A New Genus and Species of Rhynchone1Iid (Brachiopoda, Recent) from Submarine Caves in the Mediterranean Sea. Marine Ecology - P S Z N I 15(1): 77–88.

Manconi R, Ledda FD, Serusi A, Corso G, Stocchino GA (2009) Sponges of marine caves: Notes on the status of the Mediterranean palaeoendemic *Petrobiona massiliana* (Porifera: Calcarea: Lithonida) with new records from Sardinia. Italian Journal of Zoology 76: 306–315.

Muricy G, Solé-Cava AM, Thorpe JP, Boury-Esnault N (1996) Genetic evidence for extensive cryptic speciation in the subtidal sponge *Plakina trilopha* (Porifera: Demospongiae: Homoscleromorpha) from the Western Mediterranean. Marine Ecology Progress Series 138: 181–187.

Navarro-Barranco C, Guerra-García JM, Sánchez-Tocino L, García-Gómez JC (2012) Soft-bottom crustacean assemblages in Mediterranean marine caves: the cave of Cerro Gordo (Granada, Spain) as case study. Helgoland Marine Research 66: 567-576.

Pérez T, Ivanisevic J, Dubois M, Pedel L, Thomas OP, et al. (2011) *Oscarella balibaloi*, a new sponge species (Homoscleromorpha: Plakinidae) from the Western Mediterranean Sea: cytological description, reproductive cycle and ecology. Marine Ecology: An evolutionary Perspective 32: 174–187.

Pérez T, Vacelet J, Bitar G, Zibrowius H (2004) Two new lithistids (Porifera : Demospongiae) from a shallow eastern Mediterranean cave (Lebanon). Journal of the Marine Biological Association of the UK 84: 15–24.

Pisera A, Vacelet J (2011) Lithistid sponges from submarine caves in the Mediterranean: taxonomy and affinities. Scientia Marina 75: 17–40.

Radolović M, Čižmek H, Petricioli D, Bakran-Petricioli T (2006) Sponges in living communities inside the marine cave “Y”, Brbinjšćica cove, Dugi otok,. In: Besendorfer V, Klobučar G, editors. Zagreb: Proceeding of Abstracts of the 9th Croatian Biological Congress. pp. 23–29.

Rastorgueff PA, Harmelin-Vivien M, Richard P, Chevaldonné P (2011) Feeding strategies and resource partitioning mitigate the effects of oligotrophy for marine cave mysids. Marine Ecology Progress Series 440: 163–176.

Surić M (2002) Submarine karst of Croatia – evidence of former lower sea levels. Acta Carsologica 31(3): 89–98.

Surić M (2005) Submerged karst – dead or alive? Examples from the Eastern Adriatic Coast (Croatia), Geoadria 10(1): 5–19.

Surić M, Horvatinčić N, Suckow A, Juračić M, Barešić J (2005) Isotope records in submarine speleothems from the Adriatic coast, Croatia. Bulletin de la Société Géologique de France 176(4): 363–373.

Surić M, Jalžić B, Petricioli D (2007) Submerged speleothems – expect the unexpected. Examples from the eastern Adriatic coast (Croatia), Acta Carsologica 36(3): 389–396.

Surić M, Juračić M, Horvatinčić N (2004) Comparison of 14C and 230Th/234U dating of speleothems from submarine caves in the Adriatic Sea (Croatia), Acta Carsologica 33(2): 239–248.

Surić M, Lončarić R, Lončar N (2010) Submerged caves of Croatia: distribution, classification and origin. Environmental Earth Sciences 61(7): 1473–1480.

Vacelet J, Bitar G, Carteron S, Zibrowius H, Pérez T (2007) Five new sponge species (Porifera : Demospongiae) of subtropical or tropical affinities from the coast of Lebanon (eastern Mediterranean). Journal of the Marine Biological Association of the UK 87: 1539–1552.

Voultsiadou-Koukoura E, van Soest RWM, Koukouras A (1991) *Coscinoderma sporadense* sp. n. from the Aegean Sea with comments on *Coscinoderma confragosum* (Porifera, Dictyoceratida). Zoologica Scripta 20: 195–199.

Zavodnik D, Pallaoro A, Jaklin A, Kovačić M, Arko-Pijevac M (2006) A benthos survey of the Senj Archipelago (North Adriatic Sea, Croatia). Acta Adriatica 46: 3–68.

Žic V, Truesdale VW, Cukrov N (2008) The distribution of iodide and iodate in anchialine cave waters - Evidence for sustained localised oxidation of iodide to iodate in marine water. Marine Chemistry 112: 168–178.

Žuljević A, Barić Sandro A, Lemac E, Dragutin T, Zekanović H, et al. (2011) Diving in the most amazing part of the Mediterranean: Diving guide for the Šibenik – Knin County. In: Babačić Ajduk A, Škunca O (edts). Public Institution for Management of Nature Protected Areas in Šibenik Knin County pp. 128.

Websites

Caves – Karst – Springs – Mines – Subterranea. Available: <http://www.showcaves.com/english/hr/index.html> (Last accessed February 2013)

DIVING IN CROATIA (Diving Sites). Available:

<http://www.ronjenjehrvatska.com/en/home> (Last accessed February 2013)

Grotte Cosquer. Wikipédia, l'encyclopédie libre. Available:

<http://fr.wikipedia.org/wiki/Grotte_Cosquer> (Last accessed February 2013)

Project EU IPA 2007 “Identification and setting-up of the marine part of Natura 2000 network in Croatia” website. Available:

<http://www.natura2000.hr/KartaLokacija.aspx> (Last accessed February 2013)

Roniti se mora! Available:

<http://www.ronitisemora.com/lokacije/> (Last accessed February 2013)

Sea Kayak Milos. Available:

<http://www.seakayakgreece.com/virtualtour.htm> (Last accessed February 2013)

Wannadive.net – World dive site atlas. Available:

<http://www.wannadive.net/> (Last accessed February 2013)

Žuljević et al (2009) Morska bioraznolikost Biševa i JI strane otoka Visa - stručna podloga za održivo upravljanje, Project COAST, UNDP. Available: [http://issuu.com/undphr/docs/morska_bioraznolikost#embed](http://issuu.com/undphr/docs/morska_bioraznolikost#embed) (Last accessed February 2013)

Other sources

*Compilation of data from national projects/reports*

Croatia: Petra Rodic – author

France: Jean-Georges Harmelin – Station Marine d'Endoume, France; Claude Reveret – CREOCEAN

Greece: Panagiotis Dendrinos, Vasilis Gerovasileiou, Alexandros Karamanlidis – authors

Italy: Graziano Ferrari – CLEM (Centro Lubrense di Esplorazioni Marine)

Turkey: Ali Cemal Gucu – author, Serdar Bayari – International Research and Application Center for Karst Water Resources, Hacettepe University, Turkey

Cyprus: Ali Cemal Gucu – author

*Diving clubs and divers*

ALBATROS Diving, Spain: <http://www.albatros-diving.com/es/buceo-mallorca-puntos-inmersion.php>

Daris Gregory – Thessaloniki Dive Club, Greece: <http://thessalonikidiveclub.gr/core/>

Filios George – Lesvos Scuba Oceanic Centre, Greece: <http://www.lesvoscuba.gr/index.php?lang=el>

Havakis Yannis – Milos Diving Center,Greece: <http://www.milosdiving.gr/milosdiving.gr/00.html>

Ibiza Diving College, Spain: <http://www.ibiza-diving-college.com/>

Mermaid Diving Club, Turkey: <http://www.mermaiddiving.net/Divesites.asp>

Paradise Diving Malta, Malta: <http://www.paradisediving.com/cominodivesites.html>

Rivemar Dive Resort Murcia, Diving in La Azohia, Spain: <http://rivemar.com/en/dives/diving-in-la-azohia/>

Scuba Mallorca, Spain: <http://www.scubamallorca.com/es.htm>

Sofos Paris – Diving Pelion, Greece: <http://www.divingpelion.gr/>

Tritón Diving Center, Spain: <http://www.tritondivingcenter.com/en/mapa-inmersiones/>

Turtle Beach Diving Center, Greece: <http://www.diving-center-turtle-beach.com/dive_sites.php>

Vandoros George, Quality Scuba Diving Training, Greece: <http://www.george-vandoros.gr/>

West Coast Divers Mallorca, Spain: [http://www.divinginmajorca.com/divespots.html#portopi](http://www.divinginmajorca.com/divespots.html#portopi)

Antoniadis Germanos – Diver, Greece

Antonopoulos Christos – Diver, Greece

Despotopoulos Antonis – Diver, Greece

Giourgis Ektoras – Diver, Greece

Mavidis George – Diver, Greece

Poursanidis Dimitris – Marine Ecologist/Diver, Greece

*Caving clubs/cavers/speleologists and cave divers*

Boutaras Komninos – SELAS Club, Greece

Ceylan Hande – MADAG-METU Subaqua Society-Cave Diving Research Group, Turkey

Fotinakis Kostas – Hellenic Speleological Society, Department of Crete, Greece

Trimmis Konstantinos – Hellenic Speleological Society, Department of North Greece, Greece

Lazaridis George – Hellenic Speleological Society, Department of North Greece, Greece

Mavroudi Nektaria – SPOK Clud, Greece

Sfakianakis Dimitris – SPOK Club, Greece

Theodosiadis Thomas – SPELEO Club, Greece

Zacharias Stelios – SELAS Club, Greece
